# Spatial epidemiology of *Tabanus* (Diptera: Tabanidae) vectors of *Trypanosoma*

**DOI:** 10.1186/s13071-025-06708-z

**Published:** 2025-04-03

**Authors:** Roberta Marques, Daniel Jiménez-García, Luis E. Escobar, Tiago Kütter Krolow, Rodrigo Ferreira Krüger

**Affiliations:** 1 Programa de Pós-Graduação em Parasitologia, Departamento de Microbiologia e Parasitologia, Instituto de Biologia, Universidade Federal de Pelotas, Campus Universitário, s/n, Capão do Leão, Rio Grande do Sul, CEP: 96010-900 Brazil; 2 Laboratorio de Biodiversidad, Centro de Agroecología y Ambiente, Instituto de Ciencias de la Benemérita Universidad Autónoma de Puebla, Puebla, México; 3 Laboratorio Nacional CONAHCYT de Biología del Cambio Climático, Veracruz, México; 4 Department of Fish and Wildlife Conservation, Virginia Tech, Blacksburg, VA USA; 5 Laboratório de Entomologia, Coordenação de Ciências Biológicas, Universidade Federal do Tocantins, Caixa Postal: 136, Rua 03, Qd 17 s/n, Jardim dos Ipês, Porto Nacional, Tocantins, CEP 77500-000 Brazil; 6 Laboratório de Ecologia de Parasitos e Vetores, Departamento de Microbiologia e Parasitologia, Instituto de Biologia, Universidade Federal de Pelotas, Campus Universitário, s/n, Capão do Leão, Rio Grande do Sul, CEP: 96010-900 Brazil

**Keywords:** Ecological niche modeling, Illness risk, Neotropics, Horse fly, Trypanosomiasis

## Abstract

**Background:**

*Trypanosoma* are protozoa parasites that infect animals and can cause economic losses in cattle production. *Trypanosoma* live in the blood and are transmitted by hematophagous insects, such as flies in the genus *Tabanus.* Using ecological niche models, we explored the current geography of six common *Tabanus* species in Brazil, which are considered vectors of *Trypanosoma vivax* and *Tr. evansi* in the Neotropics.

**Methods:**

We used georeferenced data and biotic and abiotic variables integrated using a fundamental ecological niche modeling approach. Modeling results from six *Tabanus* species were used to identify risk areas of *Trypanosoma* transmission in Latin America accounting for area predicted, landscape conditions, and density of livestock. We performed Jaccard, Schoener, and Hellinger metrics to indicate the ecological niche similarities of pairs of *Tabanus* species to identify known and likely vectors overlapping in distribution across geographies.

**Results:**

Our results revealed significant ecological niche similarities for two *Tabanus* species (*T. pungens* and *T. sorbillans*), whereas *T. triangulum* and *T. importunus* have low ecological similarity. Ecological niche models predicted risk of *Trypanosoma* transmission across Neotropical countries, with the highest risk in southern South America, Venezuela, and central Mexico.

**Conclusions:**

More than 1.6 billion cattle and 38 million horses are under a threat category for infection risk. Furthermore, we identified specific areas and livestock populations at high risk of trypanosomiasis in Latin America. This study reveals the areas, landscapes, and populations at risk of *Trypanosoma* infections in livestock in the Americas.

**Graphical Abstract:**

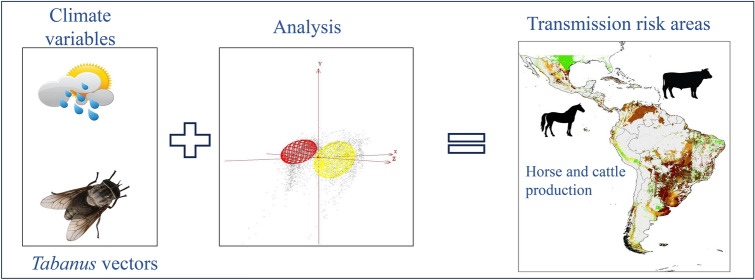

**Supplementary Information:**

The online version contains supplementary material available at 10.1186/s13071-025-06708-z.

## Background

Tabanidae is a family commonly known as "horse flies" and “deer flies,” of which *Tabanus* is the most speciose genus with a worldwide distribution, with approximately 1440 species described globally [1]. *Tabanus* males are phytophagous while females are typically hematophagous for oocyte maturation, and after the ovogenesis process, they change to phytophagous [2]. During the feeding process, female *Tabanus* flies can transmit pathogens, such as *Trypanosoma evansi* and *Tr. vivax*, to their host [3–5]. In the Neotropical region (tropics in the Americas), *Trypanosoma* parasites cause a disease termed “surra” and “trypanosomiasis” in domestic animals. *Trypanosoma* parasites have been found in domestic animals, including dogs [6, 7] and livestock (mainly in cattle and horses) [8], and in wildlife, such as capybaras, coatis, marsupials, rodents, bats, armadillos, deer [8, 9], and native camelids [10].

In the Neotropical region, the genus *Tabanus* is represented by 200 species [11], many of which are recognized as principal vectors for zoonotic pathogens [3, 12–15]. Protozoa can be transmitted mechanically by female *Tabanus* adapted to hematophagy in horses, cattle, and wild animals [16]. In the Neotropical region, there are about 190 species of the genus *Tabanus* [11], and at least five of them could be identified as mechanical vectors (pathogen transmission between host to host) of *Tr. evansi* and *Tr. vivax*, including *Tabanus claripennis*, *Tabanus importunus*, *Tabanus nebulosus*, *Tabanus pungens*, *Tabanus sorbillans*, and *Tabanus triangulum* [3, 12–15]. These *Tabanus* species have important characteristics that make them effective vectors of pathogens. For instance, their large oral proboscis retains significant amounts of blood, and they are persistent biters; these features facilitate the transfer of pathogens during blood feeding [11, 17]. Additionally, these *Tabanus* species are present in large numbers in production herds and wild animal populations, such as capybaras and coatis, which are considered reservoirs of *Trypanosoma* species [16–18]. These six species have been identified as potential mechanical vectors for *Tr. vivax* and *Tr. evansi* [16, 17, 19]. These six *Tabanus* species are abundant in natural areas and farmland where domestic animals and wildlife reservoirs of *Trypanosoma* species co-occur [18–20]. Environmental factors, such as climate, are known to restrict the abundance and distribution of *Tabanus* species [19, 21, 22].

Animal trypanosomiasis, mainly bovine trypanosomiasis caused by *Trypanosoma vivax*, is indeed present in the Americas, and certain areas can be considered hotspots due to the prevalence and impact of the disease [23]. Although the relationships between climate and *Tabanus* are well documented [11, 24], little is known about the hotspots of trypanosomiasis transmission across the Americas. Nevertheless, inferences can be made that outbreaks are closely associated with the presence and abundance of horseflies *Tabanus* during specific times of the year and in particular locations [25–27]. Ecological niche modeling has been used to assess the distributional ecology and spatial epidemiology of pathogens [28], vectors [29–31], and disease reservoirs [32]. The aims of this study were to (i) asses geographic and environmental ranges of six *Tabanus* species considered disease *Trypanosoma* vectors in the Neotropics, (ii) determine niche overlap among *Tabanus* species, and (iii) elucidate the role of grassland and livestock density on the risk of trypanosomiasis transmission to livestock in Latin America.

## Methods

### Selected *Tabanus* species

We selected *Tabanus* species according to literature data indicating the *Tabanus* potential to transmit *Trypanosoma* based on morphological, behavioral, and epidemiological information [3]. *Tabanus importunus* is a species with high vectorial potential, mentioned since the first investigations carried out by the Brazilian Dr. Adolph Lutz (1907) [33], which indicated it as a mechanical vector of *Tr. evansi* during a study carried out on Marajó Island in the state of Pará. Due to the population peak observed in the Brazilian Pantanal, a site with an outbreak of *Tr. vivax*, where this species was found to be the most abundant and therefore indicated as a protozoan vector [26, 32, 34]. *Tabanus nebulosus* is considered a "good vector" because it presents a time of blood feeding between 1 and 10 min, and it has been proven experimentally that between 17 and 19 individuals are enough for the transmission of *Tr. vivax* [12]. The number of flies of this species is not indicated as a single characteristic for the incidence of trypanosomiasis in farms [12]. In an epidemiological study with bovine herds conducted by Martins et al. [26], the most abundant species were: *T. sorbillans*, *Tabanus palpalis*, *T. claripennis*, and *T. importunus*. The authors associated the outbreak of trypanosomiasis with the horse flies’ population peak. The epidemiological studies related to protozoa *Tr. vivax* and *Tr. evansi* considered the abundance of *Tabanus* an important driver in the transmission of these parasites. Therefore, *T. triangulum* can be indicated as a possible mechanical vector of *Trypanosoma* in southern Brazil because it presents great abundance compared to the other *Tabanus* collected in this area [32, 35].

In addition to these species, epidemiological studies and Tabanidae collected in the Neotropical region found that *Tabanus occidentalis* was the most abundant species during collection, compared to other captured *Tabanus* species, suggesting that *T. occidentalis* may play an important role in transmission of *Trypanosoma* [26, 32, 34]. Nevertheless, *T. occidentalis* is a species that seems to have taxonomic problems [36, 37]. *Tabanus occidentalis* presents cryptic species and several subspecies and was removed from our study.

### Occurrence data

Neotropical distributional data of vector *Tabanus* species (*T. claripennis*, *T. importunus*, *T. nebulosus*, *T. pungens*, *T. sorbillans*, and *T. triangulum*) were obtained manually from the Entomology Collection of the University of Tocantins, Entomology Collection of the University of Pará, and the Entomology Collection of the National Institute of Amazon Research. Data were also collected digitally from the Global Biodiversity Information Facility [38–43] and Species Link [44]. Additional records were recovered through the review of publications on the species available in Web of Science, Google Scholar, and SciELO (Additional file 1). We used all publications with the keywords: “*Tabanus claripennis*,” “*Tabanus importunus*,” “*Tabanus nebulosus*,” “*Tabanus pungens*,” “*Tabanus sorbillans*,” and “*Tabanus triangulum*” published during 1950 and 2020, retaining records with geographic coordinates. To avoid synonymy errors, species names were confirmed in the Catalogue of Neotropical Diptera, Tabanidae [11]*.* We used Google Earth software to determine geographical coordinates from records recovered from literature references with information of the capture location, keeping only information at the municipality or locality level, considering an uncertainty < 5 km^2^. We reduced bias effects (oversampled areas) and spatial correlation in occurrence data using spThin R package to filter occurrence records based on distance [45]. Distance to remove records was set according to the variable’s resolution used for model construction (~ 4.5km^2^), which resulted in a final occurrence dataset of each *Tabanus* species (Additional file 2).

### Climate data

We used the 19 climatic variables data from WorldClim Global Climate Database 1.4 at 2.5 min (http://www.worldclim.org, [46]). We excluded variables 8, 9, 18, and 19, because they represent spatial artifacts [47], and instead used the remaining 15 variables to perform a principal component analysis (PCA) in NicheA software [48]. For model calibration we used the first six principal components (PC), which summarized > 99% of the variance from the original variables. We used an **M** hypothesis [49] to propose the likely accessible area of these species via 100-km buffers around each occurrence, which aimed to reduce the background effect on model calibration and selection or overfitting [50] (Additional file 3).

### Ecological niche models

Ecological niche models were calibrated and evaluated using MaxEnt 3.4.1 [51] via kuenm R package [52] using as predictors the first six PCs and randomly 50% of occurrence data for calibration and the remaining 50% for model evaluation [53]. We explored different parameters for candidate models (linear “l,” quadratic “q,” and product “p”) with combinations of response features (l, q, lq, lp, qp, lqp) and different regularization multiplier values (0.1, 0.3, 0.5, 0.7, 1, 3, 5, 7, 10) as a means to reconstruct the species fundamental niche [54]. Final parameters were chosen from all candidate models through their significance, performance, and complexity [52]. The final models were selected based on three criteria: (i) significance indicated by partial ROC [54, 55], (ii) performance delimited for omission rate at 5%, and (iii) model complexity and good fit to the data, according to the Akaike information criterion with a correction for small samples (AICc) [56]. Final models were summarized via 10 bootstrap replicates. The best model for each species was projected to the Neotropics. To identify extrapolative areas, we compare calibration areas and projection areas using MOP analysis, which indicates regions with extrapolative risk [57].

### Niche ellipsoids and overlap

We used NicheA software [48] to generate niche ellipsoid models from the occurrence data of each *Tabanus* species as a proxy of fundamental ecological niches. We explored species distributions in environmental space linked to the geographic distribution as a proxy of the Hutchinson’s duality concept of the relationship between environmental and geographic space [48]. We built the environmental space in NicheA using the first three PCs and filtered occurrence data. We estimated the ellipsoid volume for each species, a measure of ecological generalist (broad niche or large volume) vs. specialist (narrow niche or low volume) species, and calculated the niche overlap for pairs of species.

### Niche similarities

We quantified niche similarity on pairs of species using Schoener’s *D* and Hellinger’s *I* statistics, where values of 0 denoted niche models having no overlap and 1 denoting complete niche overlap [56]. Niche similarity measurements were done using ENM Tools v.1.3 [56], which analyzes niche similarity in geographic space by comparing one species to another regarding the amount and distribution of suitable pixels. Complementarily, we measured niche similarity in environmental space using the Jaccard index [58], estimated with of volume, and ellipsoid overlap, estimated in NicheA, where values of 0 denoted niche models having no overlap and 1 denoting complete niche overlap.

### Risk mapping

Final Maxent models were binarized by threshold values equivalent to an omission error of *E* = 0.05. Subsequently, binary results were stacked for the six *Tabanus* species to indicate an ensemble model of areas of potential species distribution. Publicly available Neotropical grassland area data [59] were used to match regions climatically suitable to *Tabanus* species and with grass available for livestock. The resulting map was a proxy of areas of vector-borne disease risk for livestock.

The risk map denoted areas suitable to *Tabanus* species based on landscape (i.e. grassland) and abiotic conditions (i.e. climatic). To determine the capacities of our *Tabanus* risk map to inform disease transmission risk to cattle and horses, we fitted a linear model (estimated using OLS) to assess the association between the livestock density and *Tabanus* risk map. Because no a priori information was available, we assumed that patterns of association should be able to be captured under a linear relationship. Data of livestock density were obtained from Harvard Dataverse at 5 min resolution, including densities of cattle [60] and horses [61]. Standardized parameters of the linear model were obtained by fitting the model on a standardized version of the dataset. The 95% confidence intervals and *p*-values were computed using a Wald t-distribution approximation.

## Results

The six *Tabanus* species studied were reported in the whole Neotropical region, together covering most Latin American countries (Additional file 3a and 3b). We recovered 622 filtered occurrence records for the six *Tabanus* species' ecological niche models (Additional file 2). *Tabanus importunus* presented the highest number of occurrences (*n* = 149), and the smallest amount was recorded for *T. nebulosus* (*n* = 60). We found that the six *Tabanus* species had broad distributions along Latin American countries in the Neotropics, including Brazil, Argentina, Paraguay, and Bolivia, while *Tabanus triangulum* was the species most geographically restricted, occurring only in Brazil, Bolivia, Argentina, Paraguay, and Uruguay.

**Fig. 1:**
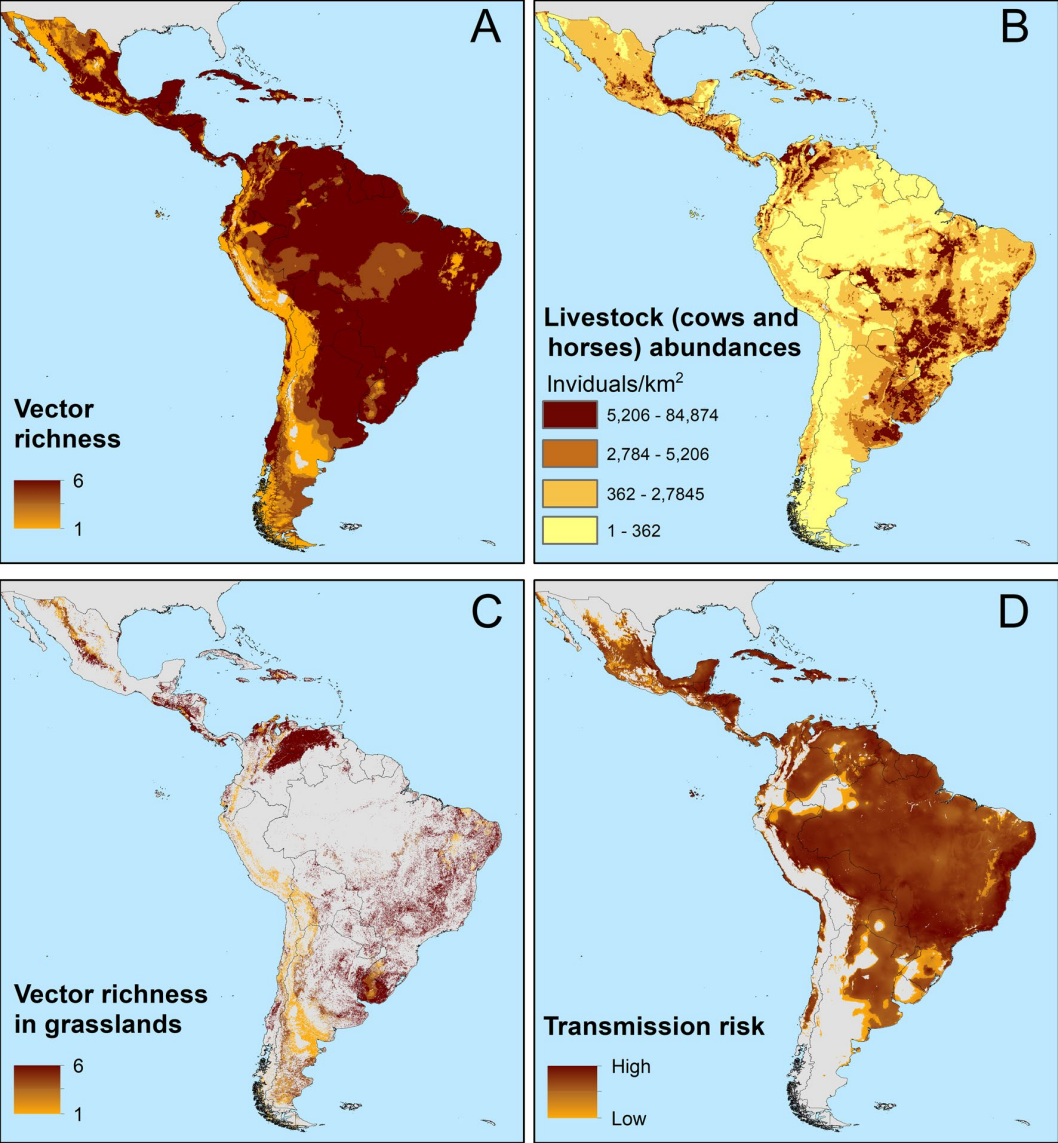
Risk maps of Tabanus-borne trypanosomiasis in the Neotropics. **A** Potential risk areas for distribution of *Tabanus* species from climate-based ecological niche models. Colors show the high (dark brown) and low (yellow) areas potentially suitable to richness of six *Tabanus* species. **B** Grassland natural areas [59] and cattle and horse areas [60, 61] in the Neotropical region. **C** Estimated distribution of *Tabanus* species in natural grassland areas. **D** Risk map showing the correlation between *Tabanus* species richness and livestock density denoting areas of high (dark brown) and low (yellow) disease transmission risk

We calibrated and evaluated 54 candidate models for each *Tabanus* species for a total of 324 ecological niche models covering diverse parameter and predictor combinations (Additional file 4). Model calibration experiments for all *Tabanus* species retrieved one final best model according to the predictive performance and fit metrics, except for *T. nebulosus*, for which three best models were identified (Additional file 4 and 5). The best models representing species' fundamental ecological niches and the average ensemble model for *T. nebulosus* were projected to the Neotropical region to identify areas potentially suitable for the species across the continent (Fig. 1A).

**Fig. 2:**
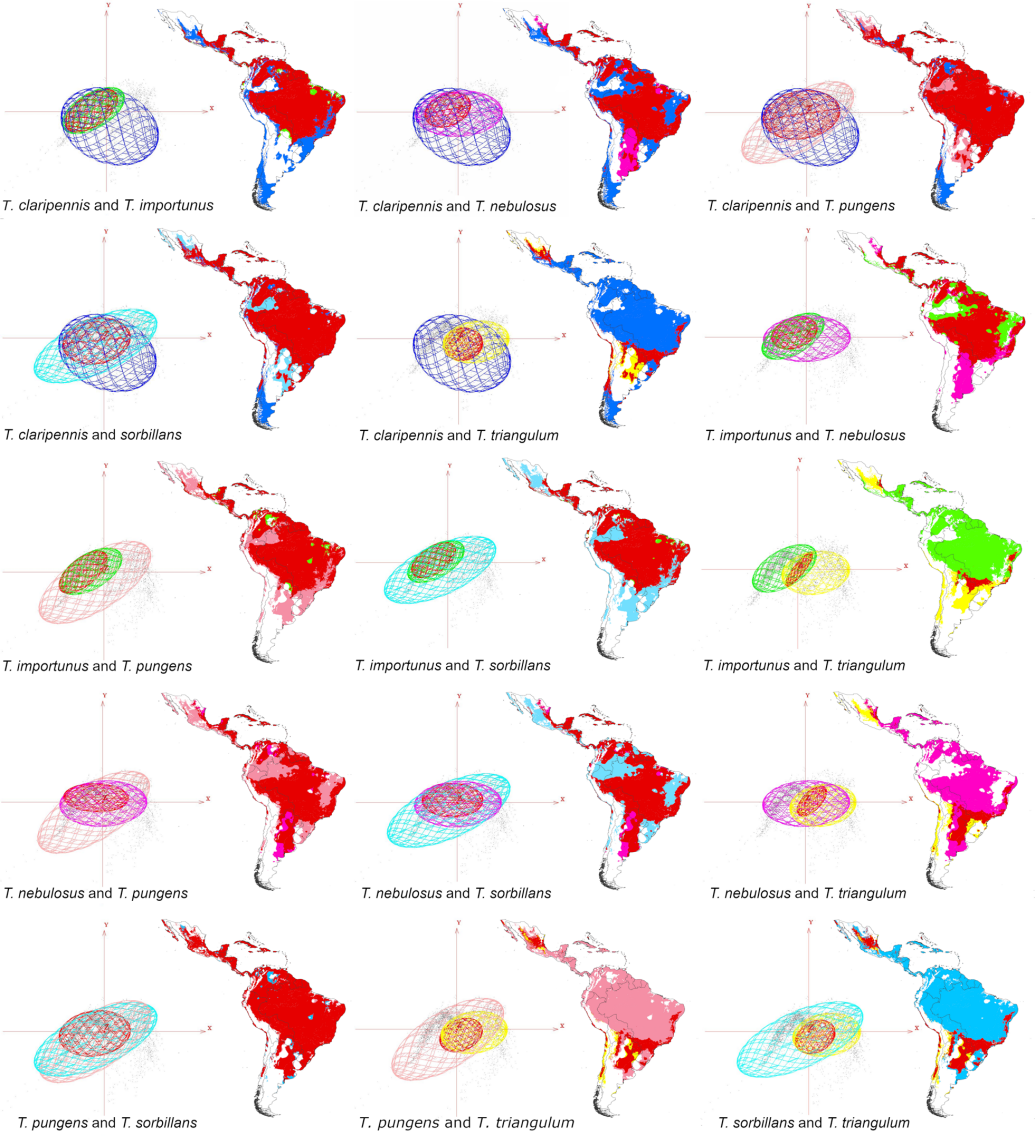
Hutchinson duality of six Tabanus species in the Netropical region. Potential distribution and niche overlap of six *Tabanus* species (dark blue: *T. claripennis*, green: *T. importunus*, pink: *T. nebulosus*, light pink: *T. pungens*, light blue: *T. sorbillans*, yellow: *T. Triangulum*, and red: niches overlap) in the environmental space available in the Neotropical region. The ellipsoids were constructed three dimensionally from the axes showing the conditions of principal component 1 (PC1: in X), principal component 2 (PC2: in Y), and principal component 3 (PC3: in Z). Maps showing the potential distribution between pairs of species and indicating the overlap between two of them (in red), according to each row and column of the matrix

Our results showed species occupying broad environmental conditions as measured by the environmental volume occupied by the occurrences. Species of large geographic distribution were ecological generalists occupying large environmental volume (i.e. *T. sorbillans* volume = 212.93, *T. nebulosus* volume = 219.90, *T. claripennis* volume = 260.81). Other species had narrow niches and restricted geographic ranges and were considered specialist species (i.e. *T. importunus* volume = 59.89, *T. triangulum* volume = 85.08, *T. pungens* volume = 103.24). In general, different *Tabanus* species occurred in disparate environmental conditions and geographies (Fig. 2).

**Fig. 3:**
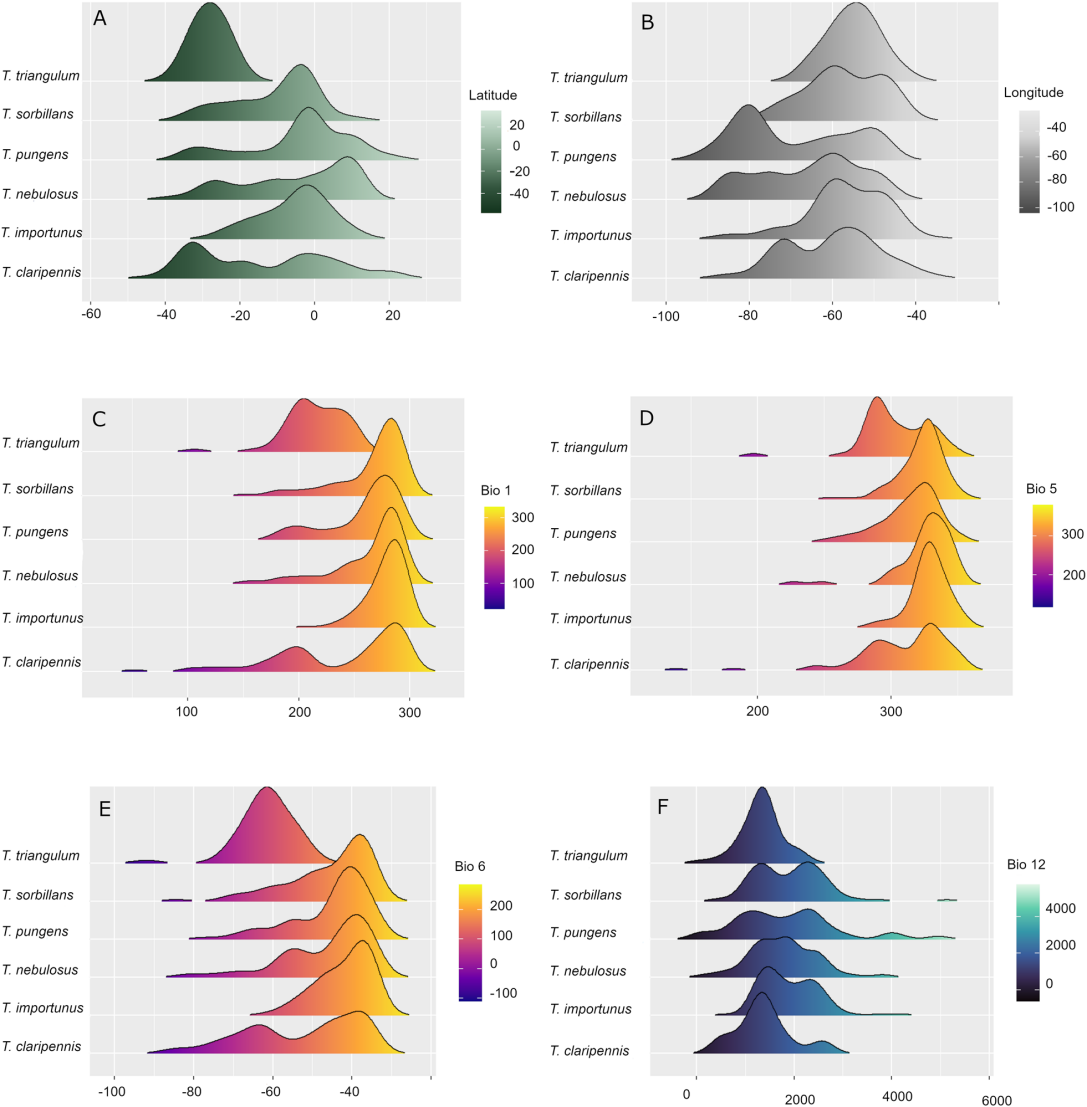
Environmental distribution of six Tabanus species in the Neotropical region. Density plots of environmental preferences of *Tabanus* species. Frequency of records of six *Tabanus* species along latitude (**A**), longitude (**B**), annual mean temperature (values: T ^O^C × 10) (**C**), maximum temperature of warmest month (values: T ^O^C × 10) (**D**), minimum temperature of coldest month (values: T ^O^C × 10) (**E**), and annual precipitation (values: mm^3^) (**F**)

Our results indicated an asymmetrical distribution of *Tabanus* species in relation to their available environment (Fig. 3). *Tabanus claripennis* had the broadest geographic and environmental distribution, occurring between latitudes 22.40°N and 45.51°S. Based on species occurrence reports, the broadest temperature tolerance was found for *T. triangulum* with temperatures ranging from –6 to 40.9 °C. The species with the narrowest temperature tolerance was *T. importunus* with temperatures ranging from 9.1 to 35.7 °C, which was also the species tolerating the warmest temperatures. Regarding humidity, *T. pungens* was the species tolerating the broadest range of annual precipitation, from 12 to 4985 mm. *Tabanus triangulum* was the species showing the narrowest range of precipitation and strong tolerance to dry conditions (75–2219 mm).

**Fig. 4:**
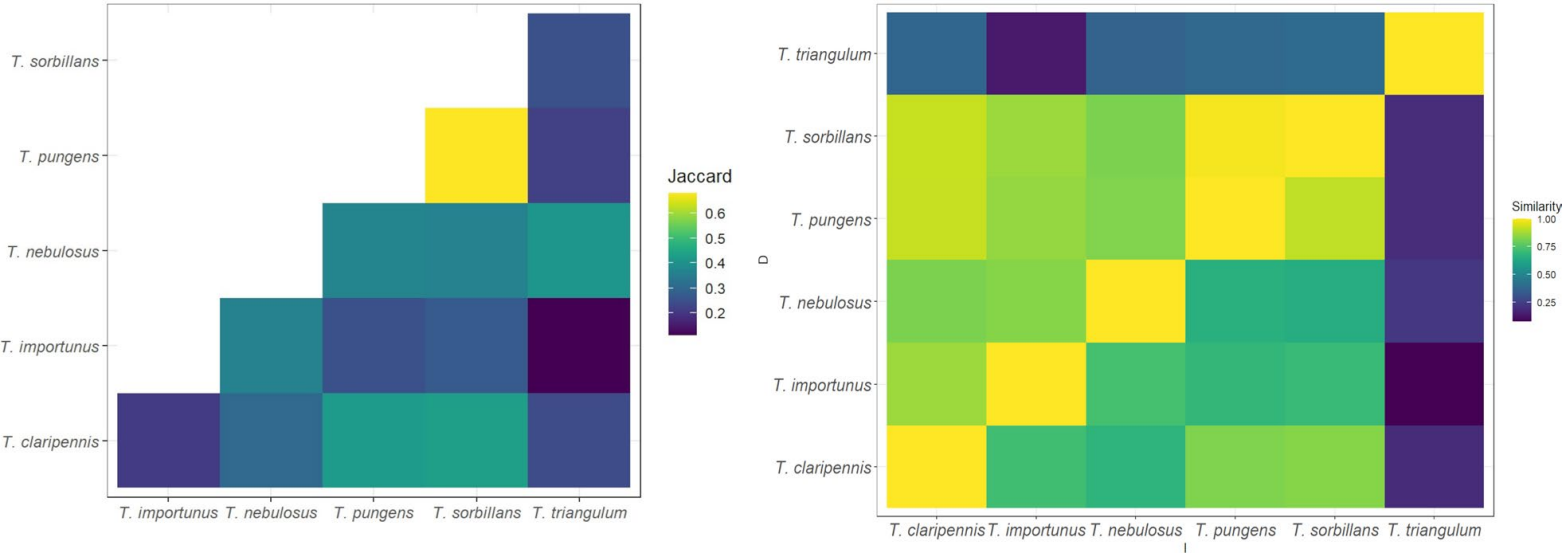
Similarity of ecological niches between pairs of Tabanus species. *A* Jaccard indexes. *B* Schoener’s (D, top of diagonal) and Hellinger’s (I, bottom of diagonal) statistics. Yellow = the greatest similarities between pairs of species; dark blues = the smallest

Niche overlap metrics revealed ecological similarity between a series of species pairs (Fig. 4). The highest niche similarity among all metrics (*I*, *D*, Jaccard indexes) was observed between *T. sorbillans* and *T. pungens* (Jaccard = 0.68, *D* = 0.91, and *I* = 0.99; Fig. 4). The lowest ecological similarity was detected in *T. triangulum*, with *T. triangulum* and *T. importunus* presenting the lowest niche similarity (Jaccard = 0.11, *D* = 0.08, and *I* = 0.14).

Our vector-borne disease risk mapping combining ecological niche models and grasslands along the whole Neotropics estimated 1.35 M km^2^ at risk of *Tabanus*-borne diseases. The total number of livestock at risk for *Tabanus*-borne parasites was 1,638,506,972 cattle and 38,861,217 horses. The regions with the highest cattle and horse densities living in hotspots of risk included eastern Argentina, Uruguay, eastern Paraguay, and central, southern, and eastern Brazil, northern Colombia, western Venezuela, and central and southern Mexico (Fig. 1B). In contrast, potential *Tabanus* distribution was not predicted along the grassland in high altitudinal regions (e.g. Andes Mountains), cold regions (Patagonia), and dry areas in the Neotropics (e.g. northern Brazil and Mexico) (Fig. 1C, D). According to our MOP analysis, MaxEnt model extrapolation is represented along different areas in the Neotropics (according with the species, Additional file 6); however, our models include low suitability in these areas, mitigated by reducing model projection in MOP-detected areas.

The regression model between livestock density and richness of *Tabanus* species (Fig. 1D) explained a statistically significant but weak proportion of variance (F(1, 1,010,059) = 65,412.40, *p* < 0.001, adj. *R*^2^ = 0.06). The model’s intercept, corresponding to livestock density = 0, was at 4.36 (95% CI [4.36, 4.36], *t*(1,010,059) = 2735.91, *p* < 0.001). Within this model the effect of livestock density on *Tabanus* occurrence was statistically significant and positive (beta = 0.0001, 95% CI [0.0001, 0.00012], *t*(1,010,059) = 255.76, *p* < 0.001; Std. beta = 0.000128, 95% CI [0.0001, 0.00012]).

## Discussion

This study estimated fundamental ecological niches, niche similarities, and geographic ranges for six *Tabanus* species implicated in pathogens transmission to cattle and horses in the Neotropics. We found that five ecological-generalist *Tabanus* species presented potential geographic distribution in areas with the highest cattle and horse production in the region. Our results generated epidemiological and ecological information about *Tabanus* in the Americas to explain likely vector-borne transmission risk of protozoan diseases, such as trypanosomiasis and anaplasmosis, and other diseases caused by virus and bacteria [3]. Results can be used to identify geographic hotspots where cattle and horses have a major risk of *Tabanus*-borne livestock diseases [62, 63]. To the best of our knowledge, this is the first study on the potential geographic distribution, environmental occupancy (mainly in highly diverse ecosystems such as Dry Chaco, Pantanal, Atlantic Forest, Humid Pampas, and Cerrado in South America; also, in the Yucatan Peninsula in Mexico), and niche similarity of *Tabanus* in the Neotropics.

### Niche breadth

Differences between the distributions of Neotropical *Tabanus* species were related to environmental heterogeneity and biological factors. That is, although *Tabanus* species occurred along diverse environmental gradients, most reports occurred under similar and consistent environmental and geographic ranges (Figs. 2 and 3). Previous research revealed linkages between environmental conditions and biological aspects of *Tabanus* related to physiology and oogenesis, especially regarding variation in temperature, humidity, and rainfall [64, 65]. Environmental variation across the study region influenced the latitudinal gradients occupied by the different species (Figs. 1A, 3, and S3). Distributional bounds of *Tabanus* are constrained by physiological tolerances, related to acclimation capacity to survive and reproduce, which in turn affect population establishment and population size [66, 67]. Also, climate change can be an important drive for these physiological changes in populations dynamics on fine [68] and regional scales[69].

### Environmental distribution

Our analyses differentiating species ecological generalists (e.g. *T. claripennis*) vs. specialists (e.g. *T. triangulum*) (Figs. 2 and 4) have direct implications for the epidemiological relevance of each species. For example, *T. importunus* was restricted to tropical regions with high temperatures such as those reported in Central America (e.g. Costa Rica and Panama) and some countries in South America (e.g. Colombia, Venezuela, Peru, Bolivia, Brazil, Paraguay, northern Argentina, French Guiana, and Guiana; Figs. 1A and 3). These findings are aligned with previous studies of *Tabanus* that found environmental constraints because of the high temperatures in dry areas of South America (e.g. Sertão Region in Brazil and dry regions in Chile; [19]).

### Seasonality

Our model ensemble denoting *Tabanus* richness restricted by grassland landscapes (Fig. 1C, D) revealed likely hotspots of disease transmission risk. Our risk model is, however, temporally static. Temporal variation in temperature and precipitation is expected to influence *Tabanus* abundance and, in turn, transmission risk. For example, in French Guiana, on the border with Brazil, *T. importunus* population peaks last between 2 and 3 months and are regulated by seasonality [70]. Similarly, a study carried out in southern Brazil indicated a strong influence of temperature and relative humidity on seasonal variation in the occurrence and abundance of *T. triangulum* [21]. During the rainy season, *T. importunus* larval stage is maintained, reaching the adult stage with the onset of the dry season [71]. In some regions of Latin America, such as Midwest Brazil, with a warm and humid climate, environmental conditions allow large populations of *T. importunus* across the year, representing up to 45% of the local *Tabanus* richness [19].

### Risk map

Our risk map combined information on *Tabanus* richness, grassland availability, and cattle and horses to identify fine-resolution transmission risk hotspots (Fig. 1B, D). A series of *Tabanus* species, including *T. importunus*, are commonly found in open landscapes dominated by pasture for cattle and horses [16, 17]. In contrast,* T. claripennis* and *T. sorbillans* prefer forested areas but can also occur in grassland [18]. The proximity between forested areas and pastures provides *Tabanus* with different food resources for adults. Male *Tabanus* feed on vegetable sap and flower nectar and pollen [72], while females also need animal blood, which may be available in wildlife and on livestock farms [3].

### Cattle density

Our ecological niche models indicated that the six *Tabanus* species studied could co-occur in the same geographic areas (Fig. 1A). This prediction is supported by previous reports in Midwest Brazil (i.e. Mato Grosso do Sul), where *T. claripennis*, *T. importunus*, *T. nebulosus*, *T. pungens*, and *T. sorbillans* coexisted in the same areas and environments [18]. Co-occurrences of *Tabanus* species have been found to be a key factor positively influencing trypanosomiasis cases [4, 5, 73–75]; almost US $5 billion in losses because of trypanosomiasis were reported in Africa [76]. This infectious disease is related to different production processes: milk production (reduction until 25% in Brazil) [77], mortality loss (almost 15% of different livestock species in India) [78], and weight loss in cattle (almost 390 g per day in Colombia) [12]. Potential distribution of *Tabanus* species is linked to the presence of the pathogens *Tr. vivax* and *Tr. evansi* in livestock [13, 18, 79]. For example, *Tabanus* presence is an important factor regarding the presence *Trypanosoma* parasites across Brazil [8, 18, 75, 79–84]. In addition, Bolivia, Colombia, Peru, and Venezuela have records of trypanosomiasis outbreaks affecting livestock, causing economic losses to farmers [12, 85–87]. In affected areas, the presence of *Tabanus* correlates with the presence of livestock and wildlife infected with *Tr. vivax* and *Tr. evansi* [20]. Almost 234,000 livestock herds are at risk in northern Brazil [88] according to the suitability of *Tabanus* species and the risk map. Wildlife has been found to represent from 25 to 45% of naturally infected *Trypanosoma* cases [13, 81]. Our results indicate that the relationship between the density of production animals and the presence of *Tabanus* species is weak and that many other factors that were not considered in the model can influence the distribution of these horseflies [18, 70]. Future research should explore other alternative landscape variables [89] with the potential to play a role in the likelihood of transmission risk (e.g. distance to rivers, socio-economic conditions, age of the landscape conversion, wildlife diversity).

### Implications

Trypanosomiasis has important implications for animal health in the Neotropical region where large outbreaks can generate devastating economic losses. For example, in the state of Espírito Santo, Brazil, a trypanosomiasis outbreak caused the death of livestock with an estimated economic loss of U$100,000 in just 1 month [90]. Incidence, distribution, and costs of trypanosomiasis in livestock could be underestimated, though. Our *Tabanus* risk maps will help address the underestimation of the burden of *Tabanus*-borne infectious diseases and can help direct vector control in localities defined as risk hotspots.

## Conclusions

Potential distribution of the six *Tabanus* species is proposed as the basis to understand how variations in abiotic (e.g. temperature, precipitation) and biotic (e.g. grassland, livestock density) factors influence the spatial epidemiology of *Tr. vivax* and *Tr. evansi*. Beyond potential distribution of *Tabanus*, their abundance could be an important variable to explain transmission risk. Further studies could combine abundance data with ecological niche models for a more accurate reconstruction of the ecology and epidemiology of trypanosomiasis. This study reconstructed the ecological niche of six *Tabanus* species to better understand their distributional ecology and to identify hotpots of trypanosomiasis transmission risk in the Neotropics, a disease of humans and animals. Here, we give some critical considerations for the epidemiology of cattle and wildlife trypanosomiasis. Climate change, physiology, and biological interactions will be the focus of the next research on *Tabanus* for the needed One Health approach.

## Supplementary Information

Supplementary Material 1. List of the references used to collect occurrence data of the six *Tabanus* species in the Neotropical region.

Supplementary Material 2. List of the occurrence records georeferenced in the Neotropical region of the six *Tabanus* species used in ecological niche modeling. These occurrences are results after the filter of the 5-km area. The coordinates are represented in decimal degrees.

Supplementary Material 3. *Tabanus* species occurrences (black points) in the Neotropical region and the 100-km buffer (M = pink circles) used in the calibration models.

Supplementary Material 4. Models calibrated and evaluated for each *Tabanus* species, according to significance, performance, and low complexity. Final models selected according to these criteria are shown in the last column.

Supplementary Material 5. Best models selected by evaluation based on pROC (statistical significance), omission rate OR (performance), and AICc (complexity). All models were calibrated and projected using principal components from 15 climatic variables from the WorldClim Global Climate Database 1.4.

Supplementary Material 6. MOP analysis of extrapolation risk from the calibration area under the Neotropical region projection. Blue areas represent levels of similarity between calibration areas and the projection areas. Red values represent strict extrapolative areas.

## Data Availability

No datasets were generated or analysed during the current study.

## References

[1] Systema Dipterorum (Tabanidae) [Internet]. [cited 2024 Sep 2]. http://www.diptera.org/. Accessed 2 Sep 2024.

[2] Foil LD, Hogsette JA. Biology and control of tabanids, stable flies and horn flies. Rev Sci Tech. 1994;13:1125–58. 7711307 10.20506/rst.13.4.821

[3] Krinsky WL. Animal disease agents transmitted by horse flies and deer flies (Diptera: Tabanidae). J Med Entomol. 1976;13:225–75. 137982 10.1093/jmedent/13.3.225

[4] Foil LD. Tabanids as vectors of disease agents. Parasitol Today. 1989;5:88–96. 15463186 10.1016/0169-4758(89)90009-4

[5] Baldacchino F, Desquesnes M, Mihok S, Foil LD, Duvallet G, Jittapalapong S. Tabanids: Neglected subjects of research, but important vectors of disease agents! Infect, Genet Evol. 2014;28:596–615. 24727644 10.1016/j.meegid.2014.03.029

[6] Da Silva A, Zanette RA, Colpo C, Santurio JM, Monteiro SG. Sinais clínicos em cães naturalmente infectados com *Trypanosoma evansi* (Kitenoplastida: Trypanosomatidae) no RS. Clin Vet. 2008;72:66–78.

[7] Franciscato C, Lopes ST dos A, Teixeira MMG, Monteiro SG, Wolkmer P, Garmatz BC, et al. Cão naturalmente infectado por *Trypanosoma evansi* em Santa Maria, RS, Brasil. Cienc Rural. 2007;37:288–91.

[8] Herrera HM, Dávila AMR, Norek A, Abreu UG, Souza SS, D’Andrea PS, et al. Enzootiology of *Trypanosoma evansi* in Pantanal. Brazil Vet Parasitol. 2004;125:263–75. 15482883 10.1016/j.vetpar.2004.07.013

[9] Silveira JAG, Rabelo ÉML, Lacerda ACR, Borges PAL, Tomás WM, Pellegrin AO, et al. Molecular detection and identification of hemoparasites in pampas deer (*Ozotoceros bezoarticus* Linnaeus, 1758) from the Pantanal Brazil. Ticks Tick-borne Dis. 2013;4:341–5. 23567028 10.1016/j.ttbdis.2013.01.008

[10] Asghari MM, Rassouli M. First identification of *Trypanosoma vivax* among camels (*Camelus dromedarius*) in Yazd, central Iran, jointly with *Trypanosoma evansi*. Parasitol Int. 2022;86:102450. 34506947 10.1016/j.parint.2021.102450

[11] Coscarón S, Papavero N. Catalogue of Neotropical Diptera - Tabanidae [Internet]. Scribd. 2023. https://www.scribd.com/document/598479338/CATALOGUE-OF-NEOTROPICAL-DIPTERA-TABANIDAE. Accessed 5 Nov 2023.

[12] Otte MJ, Abuabara JY, Wells EA. *Trypanosoma vivax* in Colombia: Epidemiology and production losses. Trop Anim Health Prod. 1994;26:146–56. 7809986 10.1007/BF02241071

[13] Silva R a. MS, Rivera Dávila AM, Seidl A, Ramirez L. *Trypanosoma evansi* e *Trypanosoma vivax*: biologia, diagnóstico e controle. [Internet]. Corumbá: Embrapa Pantanal, 2002.; 2002. http://www.alice.cnptia.embrapa.br/handle/doc/810940. Accessed 6 Nov 2023.

[14] Parra-Henao G, Alarcón-Pineda EP, López-Valencia G. Ecology and parasitological analysis of horse flies (Diptera: Tabanidae) in Antioquia. Colombia Caldasia. 2008;30:179–88.

[15] da Silva AS, Costa MM, Polenz MF, Polenz CH, Teixeira MMG, Lopes STDA, et al. First report of *Trypanosoma vixax* in bovines in the State of Rio Grande do Sul, Brazil/Primeiro registro de *Trypanosoma vivax* em bovinos no Estado do Rio Grande do Sul. Brasil Cienc Rural. 2009;39:2550–5.

[16] Barros ATM, Foil LD. The influence of distance on movement of tabanids (Diptera: Tabanidae) between horses. Vet Parasitol. 2007;144:380–4. 17112669 10.1016/j.vetpar.2006.09.041

[17] Foil LD, Gorham JR. Mechanical transmission of disease agents by arthropods. In: Eldridge BF, Edman JD, editors. Medical Entomology [Internet]. Dordrecht: Springer Netherlands; 2004. p. 461–514. 10.1007/978-94-007-1009-2_12

[18] Barros ATM. Seasonality and relative abundance of Tabanidae (Diptera) captured on horses in the Pantanal, Brazil. Mem Inst Oswaldo Cruz. 2001;96:917–23. 11685255 10.1590/s0074-02762001000700006

[19] Barros ATM, Foil LD, Vazquez SA de S. Mutucas (Diptera: Tabanidae) do Pantanal: abundância relativa e sazonalidade na sub-região da Nhecolândia. Bol Pesqui Desenvolv. 2003;48:1–20.

[20] Silva HIL. Tabanidae (Diptera) da Planície Costeira do Rio Grande do Sul [Internet]. [Pelotas, RS, Brazil]: Universidade Federal de Pelotas; 2016 [cited 2022 Feb 15]. https://wp.ufpel.edu.br/ppgent/files/2016/03/Dissertação-Helena-Iris-Leite-Tabanídeos-da-Planície-Costeira-do-RS.pdf. Accessed 15 Feb 2022.

[21] Krüger RF, Krolow TK. Seasonal patterns of horse fly richness and abundance in the Pampa biome of southern Brazil. J Vector Ecol. 2015;40:364–72. 26611972 10.1111/jvec.12175

[22] Lucas M, Krolow TK, Riet-Correa F, Barros ATM, Krüger RF, Saravia A, et al. Diversity and seasonality of horse flies (Diptera: Tabanidae) in Uruguay. Sci Rep. 2020;10:401. 31942013 10.1038/s41598-019-57356-0 PMC6962385

[23] Jones TW, Dávila AMR. *Trypanosoma vivax*—out of Africa. Trends Parasitol. 2001;17:99–101. 11228017 10.1016/s1471-4922(00)01777-3

[24] Zamarchi TB de O, Henriques AL, Krolow TK, Krüger RF, Rodrigues GD, Munari A, et al. Tabanidae (Diptera) captured on horses in Amazon Forest fragments of the state of Rondônia, Brazil. Acta Trop. 2023;237:106734. 10.1016/j.actatropica.2022.106734 36384991

[25] Silva RAMS, da Silva JA, Schneider RC, de Freitas J, Mesquita D, Mesquita T, et al. Outbreak of trypanosomiasis due to *Trypanosoma vivax* (Ziemann, 1905) in bovines of the Pantanal, Brazil. Mem Inst Oswaldo Cruz. 1996;91:561–2. 9137742 10.1590/s0074-02761996000500005

[26] Martins CF, Madruga CR, Koller WW, Araújo FR, Soares CO, Kessler RH, et al. *Trypanosoma vivax* infection dynamics in a cattle herd maintained in a transition area between Pantanal lowlands and highlands of Mato Grosso do Sul. Brazil Pesq Vet Bras. 2008;28:51–6.

[27] Neto AQ de A, Mendonça CL de, Souto RJC, Sampaio PH, Junior OLF, André MR, et al. Diagnóstico, aspectos clínicos e epidemiológicos de bovinos leiteiros naturalmente infectados por *Trypanosoma vivax* nos estados de Pernambuco e Alagoas, Brasil. Braz J Vet Med. 2019;41:e094319–e094319.

[28] Romero-Alvarez D, Peterson AT, Salzer JS, Pittiglio C, Shadomy S, Traxler R, et al. Potential distributions of *Bacillus anthracis* and *Bacillus cereus* biovar *anthracis* causing anthrax in Africa. PLoS NeglTrop Dis. 2020;14:e0008131. 10.1371/journal.pntd.0008131 PMC7082064 32150557

[29] Ceccarelli S, Balsalobre A, Susevich M, Echeverria M, Gorla D, Marti G. Modelling the potential geographic distribution of triatomines infected by *Triatoma* virus in the southern cone of South America. Parasit Vectors. 2015;8:153. 25881183 10.1186/s13071-015-0761-1 PMC4367828

[30] Alkishe AA, Peterson AT, Samy AM. Climate change influences on the potential geographic distribution of the disease vector tick *Ixodes ricinus*. PLoS ONE. 2017;12:e0189092. 29206879 10.1371/journal.pone.0189092 PMC5716528

[31] Marques R, Krüger RF, Peterson AT, de Melo LF, Vicenzi N, Jiménez-García D. Climate change implications for the distribution of the babesiosis and anaplasmosis tick vector, *Rhipicephalus* (Boophilus) *microplus*. Vet Res. 2020;51:81. 32546223 10.1186/s13567-020-00802-z PMC7298856

[32] Marques R, Krüger RF, Cunha SK, Silveira AS, Alves DMCC, Rodrigues GD. Climate change impacts on *Anopheles* (K.) *cruzii* in urban areas of Atlantic Forest of Brazil: challenges for malaria diseases. Acta Trop. 2021;224:106123. 34480869 10.1016/j.actatropica.2021.106123

[33] Lutz A. Bemerkungen über die Nomenklatur und Bestimmung der brasilianischen Tabaniden. Zentralbl Bakteriol. 1907;44:137–44.

[34] Barros T, Burger JF. Seasonal occurrence and relative abundance of Tabanidae (Diptera) from the Pantanal region. Contributions to the Knowledge of Diptera: a Collection of Articles on Diptera Commemorating the Life and Work of Graham B Fairchild. Gainesville, USA: Associated Publishers; 1999. p. 387–96.

[35] De Bassi RMA, Da Cunha MCI, Coscarón S. Estudo do comportamento de tabanídeos (Diptera, Tabanidae) do Brasil. ABPar. 2000;29:101–15.

[36] Fairchild GB. Notes on Neotropical Tabanidae (Diptera) XIX: The *Tabanus lineola* complex. Entomological Society of America; 1983.

[37] Fairchild GB, Burger JF. A catalog of the Tabanidae (Diptera) of the Americas south of the United States [Internet]. Associated Publishers; 1994 [cited 2024 Dec 12]. Available from: https://cir.nii.ac.jp/crid/1130000794284420864

[38] GBIF. *Tabanus claripennis* GBIF Occurrence Download [Internet]. 2021. https://www.gbif.org/occurrence/download/0185638-200613084148143. Accessed 9 Nov 2023.

[39] GBIF. *Tabanus importunus* GBIF Occurrence Download [Internet]. 2021. https://www.gbif.org/occurrence/download/0185640-200613084148143. Accessed 9 Nov 2023.

[40] GBIF. *Tabanus pungens* GBIF Occurrence Download [Internet]. 2021. https://www.gbif.org/occurrence/download/0185646-200613084148143. Accessed 9 Nov 2023.

[41] GBIF. *Tabanus sorbillans* GBIF Occurrence Download [Internet]. 2021. https://www.gbif.org/occurrence/download/0185650-200613084148143. Accessed 9 Nov 2023.

[42] GBIF. *Tabanus triangulum* GBIF Occurrence Download [Internet]. 2021. https://www.gbif.org/occurrence/download/0185651-200613084148143. Accessed 9 Nov 2023.

[43] GBIF. *Tabanus nebulosus* GBIF Occurrence Download [Internet]. 2023. https://www.gbif.org/occurrence/download/0185643-200613084148143. Accessed 9 Nov 2023.

[44] speciesLink: Sistema de Informação Distribuído para Coleções Biológicas [Internet]. 2020 http://splink.cria.org.br/. Accessed 4 May 2020.

[45] Aiello-Lammens ME, Boria RA, Radosavljevic A, Vilela B, Anderson RP. spThin: an R package for spatial thinning of species occurrence records for use in ecological niche models. Ecography. 2015;38:541–5.

[46] Hijmans RJ, Cameron SE, Parra JL, Jones PG, Jarvis A. Very high resolution interpolated climate surfaces for global land areas. Int J Climatol. 2005;25:1965–78.

[47] Escobar LE, Lira-Noriega A, Medina-Vogel G, Peterson AT. Potential for spread of the white-nose fungus (*Pseudogymnoascus destructans*) in the Americas: use of Maxent and NicheA to assure strict model transference. Geospat Health. 2014;9:221–9. 25545939 10.4081/gh.2014.19

[48] Qiao H, Peterson AT, Campbell LP, Soberón J, Ji L, Escobar LE. NicheA: creating virtual species and ecological niches in multivariate environmental scenarios. Ecography. 2016;39:805–13.

[49] Barve N, Barve V, Jiménez-Valverde A, Lira-Noriega A, Maher SP, Peterson AT, et al. The crucial role of the accessible area in ecological niche modeling and species distribution modeling. Ecol Modell. 2011;222:1810–9.

[50] Soberon J, Peterson AT. Interpretation of models of fundamental ecological niches and species’ distributional areas. Biodiv Inf. 2005;2:1–10.

[51] Phillips SJ, Anderson RP, Schapire RE. Maximum entropy modeling of species geographic distributions. Ecol Modell. 2006;190:231–59.

[52] Cobos ME, Peterson AT, Barve N, Osorio-Olvera L. kuenm: an R package for detailed development of ecological niche models using Maxent. PeerJ. 2019;7:e6281. 30755826 10.7717/peerj.6281 PMC6368831

[53] Peterson AT. Mapping disease transmission risk: Enriching models using biogeography and ecology [Internet]. Baltimore, US: Johns Hopkins University Press; 2014. https://muse.jhu.edu/pub/1/monograph/book/36167. Accessed 6 Nov 2023.

[54] Peterson AT, Papeş M, Soberón J. Rethinking receiver operating characteristic analysis applications in ecological niche modeling. Ecol Modell. 2008;213:63–72.

[55] Lobo JM, Jiménez-Valverde A, Real R. AUC: a misleading measure of the performance of predictive distribution models. Glob Ecol Biogeogr. 2008;17:145–51.

[56] Warren DL, Glor RE, Turelli M. ENMTools: a toolbox for comparative studies of environmental niche models. Ecography. 2010;33:607–11.

[57] Owens HL, Campbell LP, Dornak LL, Saupe EE, Barve N, Soberón J. Constraints on interpretation of ecological niche models by limited environmental ranges on calibration areas. Ecol Modell. 2013;263:10–8.

[58] Jaccard P. The distribution of the flora in the alpine zone. New Phytol. 1912;11:37–50.

[59] FAO. FAO - Food Agriculture Organization- Map Catalog [Internet]. 2023. https://data.apps.fao.org/map/catalog/srv/eng/catalog.search?id=37139#/home. Accessed 6 Nov 2023.

[60] Gilbert M, Cinardi G, Da Re D, Wint WGR, Wisser D, Robinson TP. Global cattle distribution in 2015 (5 minutes of arc) [Internet]. Harvard Dataverse; 2022. https://dataverse.harvard.edu/dataset.xhtml?persistentId=doi:10.7910/DVN/LHBICE. Accessed 9 Nov 2023.

[61] Gilbert M, Cinardi G, Da Re D, Wint WGR, Wisser D, Robinson TP. Global horses distribution in 2015 (5 minutes of arc) [Internet]. Harvard Dataverse; 2022. https://dataverse.harvard.edu/dataset.xhtml?persistentId=doi:10.7910/DVN/JJGCTX. Accessed 9 Nov 2023.

[62] Cárdenas RE, Buestán J, Dangles O. Diversity and distribution models of horse flies (Diptera: Tabanidae) from Ecuador. Ann Soc Entomol Fr. 2009;45:511–28.

[63] Dörge DD, Cunze S, Klimpel S. Incompletely observed: niche estimation for six frequent European horsefly species (Diptera, Tabanoidea, Tabanidae). Parasites Vectors. 2020;13:461. 32912281 10.1186/s13071-020-04316-7 PMC7488268

[64] Rafael JA, Charlwood JD. Physiological age, seasonal variation and daily periodicity in 4 populations of Tabanidae (Diptera) on the University Campus, Manaus. Brazil Acta Amazon. 1980;10:907–27.

[65] Roberts RH. The effect of temperature on the duration of oogenesis and embryonic development in Tabanidae (Diptera). J Med Entomol. 1980;17:8–14.

[66] Oliveira AF, Ferreira RLM, Rafael JA. Sazonalidade e atividade diurna de Tabanidae (Diptera: Insecta) de dossel na Reserva Florestal Adolpho Ducke, Manaus. AM Neotrop entomol. 2007;36:790–7. 18060307 10.1590/s1519-566x2007000500022

[67] Desquesnes M, De La Rocque S, Vokaty S. Horseflies of the Guyanas. Biology, veterinary significance and control methods [Internet]. CIRAD-EMVT; 1993. https://agritrop.cirad.fr/324171/. Accessed 27 Oct 2024.

[68] Cárdenas RE. Fine-scale climatic variation drives altitudinal niche partitioning of tabanid flies in a tropical montane cloud forest. Ecuadorian Chocó Insect Conserv Divers. 2016;9:87–96.

[69] Marques R, Alves DMCC, Vicenzi N, Krolow TK, Krüger RF. Will global warming alter the geographic distribution of *Lepiselaga crassipes* (Diptera: Tabanidae), the vector of trypanosomiasis in equines in the Neotropics? Oecol Aust [Internet]. 2017, 21. https://revistas.ufrj.br/index.php/oa/article/view/9843. Accessed 18 Dec 2024.

[70] Raymond HL. Distribution Temporelle des Principales Espèces de Taons (*Diptera: Tabanidae*) Nuisibles Au Bétail en Guyane Française. Ann Soc Entomol Fr. 1989;25:289–94.

[71] Gorayeb I de S. Tabanidae (Diptera) da Amazônia. XI - sazonalidade das espécies da Amazônia oriental e correlação com fatores climáticos. Bol Mus Para Emílio Goeldi, Zoo. 1993;9:241–81.

[72] Goldblatt P, Manning JC. The long-proboscid fly pollination system in Southern Africa. Ann Missouri Bot Gard. 2000;87:146.

[73] Desquesnes M, Biteau-Coroller F, Bouyer J, Dia ML, Foil L. Development of a mathematical model for mechanical transmission of trypanosomes and other pathogens of cattle transmitted by tabanids. Int J Parasitol. 2009;39:333–46. 18755195 10.1016/j.ijpara.2008.07.004

[74] Cadioli FA. Barnabé P de A, Machado RZ, Teixeira MCA, André MR, Sampaio PH, First report of *Trypanosoma vivax* outbreak in dairy cattle in São Paulo state, Brazil. Rev Bras Parasitol Vet. 2012;21:118–24. 10.1590/s1984-29612012000200009 22832751

[75] Paiva F, Lemos RAA, Nakazato L, Mori AE, Brum KB, Bernardo KC. *Trypanosoma vivax* em bovinos no Pantanal do Estado do Mato Grosso do Sul, Brasil: I.-Acompanhamento clínico, laboratorial e anatomopatológico de rebanhos infectados. Brazil J Vet Parasitol. 2000;9:135–41.

[76] Angara T-E.E AT-EE, Ismail A. A IAA, Ibrahim A.M IAM. An overview on the economic Impacts of animal trypanosomiasis. GRA. 2012;3:275–6.

[77] Barbosa JC, Bastos TSA, Rodrigues RA, Madrid DMC, Faria AM, Bessa LC, et al. Primeiro surto de tripanossomose bovina detectado no estado de Goiás. Brasil Ars Vet. 2015;31:100.

[78] Kumar R, Jain S, Kumar S, Sethi K, Kumar S, Tripathi BN. Impact estimation of animal trypanosomosis (surra) on livestock productivity in India using simulation model: Current and future perspective. Vet Parasitol Reg Stud Reports. 2017;10:1–12. 31014579 10.1016/j.vprsr.2017.06.008

[79] Dávila AMR, Herrera HM, Schlebinger T, Souza SS, Traub-Cseko YM. Using PCR for unraveling the cryptic epizootiology of livestock trypanosomosis in the Pantanal. Brazil Vet Parasitol. 2003;117:1–13. 14597273 10.1016/j.vetpar.2003.08.002

[80] Batista JS, Riet-Correa F, Teixeira MMG, Madruga CR, Simões SDV, Maia TF. Trypanosomiasis by *Trypanosoma vivax* in cattle in the Brazilian semiarid: description of an outbreak and lesions in the nervous system. Vet Parasitol. 2007;143:174–81. 16965857 10.1016/j.vetpar.2006.08.017

[81] Olifiers N, Jansen AM, Herrera HM, Bianchi R de C, D’Andrea PS, Mourão G de M, et al. Co-Infection and wild animal health: Effects of trypanosomatids and gastrointestinal parasites on coatis of the Brazilian Pantanal. PLOS ONE. 2015;10:e0143997. 10.1371/journal.pone.0143997 PMC4678147 26657699

[82] Lopes FC. Infecção natural e experimental de *Trypanosoma vivax* em rebanhos leiteiros [Internet]. [Mossoró, Rio Grande Do Norte]: Universidade Federal Rural Do Semi-Árido; 2015. https://sucupira.capes.gov.br/sucupira/public/consultas/coleta/trabalhoConclusao/viewTrabalhoConclusao.jsf?popup=true&id_trabalho=2730060. Accessed 6 Nov 2023.

[83] Rodrigues A, Fighera RA, Souza TM, Schild AL, Soares MP, Milano J, et al. Surtos de tripanossomíase por *Trypanosoma evansi* em eqüinos no Rio Grande do Sul: aspectos epidemiológicos, clínicos, hematológicos e patológicos. Pesq Vet Bras. 2005;25:239–49.

[84] Zanette RA, Silva AS da, Costa MM da, Monteiro SG, Santurio JM, Lopes ST dos A. Ocorrência de *Trypanosoma evansi* em eqüinos no município de Cruz Alta, RS, Brasil. Cienc Rural. 2008;38:1468–71.

[85] Silva RAMS, Egüez A, Morales G, Eulert E, Montenegro A, Ybañez R, et al. Bovine Trypanosomiasis in Bolivian and Brazilian lowlands. Mem Inst Oswaldo Cruz. 1998;93:29–32. 9698839 10.1590/s0074-02761998000100006

[86] Mekata H, Konnai S, Witola WH, Inoue N, Onuma M, Ohashi K. Molecular detection of trypanosomes in cattle in South America and genetic diversity of *Trypanosoma evansi* based on expression-site-associated gene 6. Infect, Genet Evol. 2009;9:1301–5. 19664722 10.1016/j.meegid.2009.07.009

[87] Ramírez-Iglesias JR, Eleizalde MC, Reyna-Bello A, Mendoza M. Molecular diagnosis of cattle trypanosomes in Venezuela: evidences of *Trypanosoma evansi* and *Trypanosoma vivax* infections. J Parasit Dis. 2017;41:450–8. 28615858 10.1007/s12639-016-0826-x PMC5447603

[88] Nery C. Herds and value of products of animal origin hit record in 2022 [Internet]. Agência de Notícias - IBGE. 2023. https://agenciadenoticias.ibge.gov.br/en/agencia-news/2184-news-agency/news/37941-rebanhos-e-valor-dos-principais-produto-de-origem-animal-foram-recordes-em-2023. Accessed 19 Dec 2024.

[89] de Oliveira Zamarchi TB, Henriques AL, Krolow TK, Krüger RF, Rodrigues GD, Guimarães AM, et al. Diversity and seasonality of horse flies (Diptera: Tabanidae) in Amazon forest fragments of Monte Negro, Rondônia, Western Amazon. Parasitol Res. 2024;123:288. 39093485 10.1007/s00436-024-08292-0

[90] PRODEST. Idaf [Internet]. Idaf. 2023. https://idaf.es.gov.br. Accessed 4 Nov 2023.

