# Supplementary material for: Spatial epidemiology of Tabanus (Diptera: Tabanidae) vectors of Trypanosoma
Source: Parasit Vectors. 2025 Apr 3;18:128. doi: 10.1186/s13071-025-06708-z (PMC11969902; doi:10.1186/s13071-025-06708-z)
Supplement: Supplementary file 1 — Supplementary Material 1. List of the references used to collect occurrence data of the six Tabanus species in the Neotropical region. [file 13071_2025_6708_MOESM1_ESM.doc]

Additional file 1. List of the references used to collect occurrence data of the six *Tabanus* species in the Neotropical region.

Barbosa MGV, Henriques AL, Rafael JA, Fonseca CRV. Diversidade e similaridade entre habitats em relação às espécies de Tabanidae (Insecta: Diptera) de uma floresta tropical de terra firme (Reserva Adolpho Ducke) na Amazônia Central, Brasil. 2005; 18:251-66.

Barros ATM. Seasonality and relative abundance of Tabanidae (Diptera) captured on Horse in the Pantanal, Brazil. Memórias do Instituto Oswaldo Cruz. 2001; 96:917-23.

Barros ATM, Foil LD, Vazques SAS. Mutucas (Diptera: Tabanidae) do Pantanal: abundância relativa e sazonalidade na sub-região da Nhecolândia. Embrapa, Boletim de Pesquisa e Desenvolvimento. 2003; 48: 1-20.

Barros, A. T. M., Foil, L. D. 2007. The influence of distance on movement of tabanids (Diptera: tabanidae) between horses. Veterinary Parasitology, 144 (3-4), pp. 380-384.

Bassi RMA, Cunha MCI, Coscarón S. Estudo do comportamento de tabanídeos (Diptera, Tabanidae) do Brasil. Acta Biológica Paranaense. 2000; 29:101-115.

Benchimol, J. L., Sá, M. R. 2005. Adolpho Lutz Obra Completa, v2. Ed. FIOCRUZ, Rio de Janeiro, Brasil, 704p. Tabanídeos do Brasil e de algunos Estados vizinhos. pp.419-452.

Bequaert, J. C., Renjifo-Salcedo, S. 1946. The Tabanidae of Calombia. Psyche, 53 (3-4), pp. 52-88.

Cárdenas RE, Buestán J, Dangles O. Diversity and distribution models of horse flies (Diptera: Tabanidae) from Equador. Annales de la Société Entomologique de France. 2007. doi: 10.1080/00379271.2009.10697633.

Chainey, J. E., Hall, M. J., Aramayo, J. L. B., Bettella, P. 1994. A preliminar checklist and key to the gerena and sungenera of Tabanidae (Diptera) of Bolivia with particular reference to Santa Cruz Department. Memórias do Instituto Oswaldo Cruz, 89(3), pp.321-345.

Coscarón S. Notas sobre tabanidos argentino (Insecta: Diptera). VII Los tabanos del Delta del Parana. Physis. 1968; 76: 39-53.

Coscarón S. Notas sobre Tabanidos argentinos XV. El gênero Tabanus Linnaeus (Diptera-Insecta). Obra del Centenario del Museu de La Plata. 1979; 6:251-73.

Coscarón S, González CR. Tabanidae de Chile: Lista de espécies y clave para los generos conocidos de Chile (Diptera: Tabanidae). Acta Entomológica Chilena. 1991; 16:125-50.

Coscarón S. Clave ilustrada de larvas y pupas de Tabanidae de Argentina (Diptera, Insecta). In Salomón, O. D. (ed.). Actualizaciones en artropodología sanitaria argentina. Ed. Mundo Sano (Serie Enfermedades Transmisibles, Publicación monográfica 2), Buenos Aires. 2002. pp. 11-21.

Coscarón S, Papavero N. Catalogue of Neotropical Diptera. Tabanidae. Neotropical Diptera 16. 2009. pp. 1-199.

Duda O. Die Ausbeute der Deustchen Chaco-Expedition 1925/26 (Diptera). X Chloropidae. Konowia. 1929; 8:165-93.

Dutra RRC, Marinoni RC. Insetos capturados com armadilha Malaise na Ilha do Mel, Baía de Paranaguá, Paraná, Brasil. II Tabanidae (Diptera). Revista Brasileira de Zoologia. 1994; 11:247-56.

Fairchild GB. Notes on neotropical Tabanidae (Diptera) from Panama VII. The subgenus Neotabanus ad. Lutz. Annals Entomological Society of America. 1942; 35:153-82.

Fairchild GB. The seasonal distribution of some Tabanidae (Diptera) in Panama. Annals Entomological Society of America. 1942; 35:85-91.

Fairchild GB, Ortiz I. Algunos Tabanidae del Bajo Orinoco, Venezuela. Novedades Cientificas – Contribuiciones Ocasionales del Museo de Historia Natural la Salle. 1955; 16:2-7.

Fairchild, GB. Insecta Amapaensia – Diptera: Tabanidae. Studia Entomologica. 1961; 4:433-48.

Fairchild GB. Notes on neotropical Tabanidae (Diptera) X. The species described by J.R. Schiner and others. Pacific Insects. 1967; 9:243-56.

Fairchild GB. Notes on neotropical Tabanidae (Diptera) XX. The larger species of Tabanus of eastern South America. Contributions of the American Entomological Institute. 1984; 21:1-50.

Ferenc SA, Raymond HL, Lancelot R, Courtney CH. Mechanical transmission of South American *Trypanosoma vivax* by the tabanid *Cryptotylus unicolor*. Université de Floride, Gainesville. 1988.

Fernández LD, González LR, Alonso CS. Nueva relación de insectos de importância médica para la cayeria noroccidental de Camaguey. Estudio preliminar. Revista Cubana de Medicina Tropical. 1997; 49:1-4.

Ferreira RLM, Henriques AL, Rafael JA. Activity of Tabanids (Insecta: Diptera: Tabanidae) Attacking the reptiles caiman crocodilos (Linn.) (Alligatoridae) and Eunectes murinus (Linn.) (Boidae), in the Central Amazon, Brazil. Memórias do Instituto Oswaldo Cruz. 2002. doi:http://dx.doi.org/10.1590/S0074- 02762002000100024.

Ferreira-Keppler R, Rafael JA, Guerrero JCH. Sazonalidade e uso de ambientes por espécies de Tabanidae (Diptera) na Amazônia Central, Brasil. Neotropical Entomology. 2010; 39:645-54.

Goméz ZT, Ríos MV, Gorayeb IS. Lista preliminar de tabánidos (Diptera: Tabanidae) del Noroccidente de Guárico y Sur de Aragua, Venezuela. Entomotropica. 2004; 19:59-63.

Gorayeb, I. S. 2000. Tabanidae (Diptera) da Amazônia. XVI - atividade diurna de hematofagia de espécies da Amazônia Oriental, em áreas de mata e pastagens, correlacionada com fatores climáticos. Boletim do Museu Paraense Emílio Goeldi, série Zoologia, 16 (1), pp. 23-63.

Gorayeb IS, Ribeiro JMF. Tabanidae (Diptera) da Amazônia XVII. Deslocamento à hospedeiros determinados por marcação e recaptura. Boletim do Museu Paraense Emílio Goeldi. 2001; 17:69-100.

Guimarães RR, Guimarães Júnior RR, Harlan-Rodrigues RS, Guimarães RR, Carvalho RW. Checklist and notes on behavior of horse flies Marambaia Island , Rio de Janeiro, Brazil, with new records for the State. Entomo Brasilis. 2016; 9,2:73-80.

Henriques, A. L. 1993. A coleção de Tabanidae (Insecta: Diptera) do museu paraense Emílio Goeldi, Belém, Pará, Brasil. Goeldiana Zoologia, 20(25), pp.1-23.

Henriques, A. L. 1995. A coleção de Tabanidae (Insecta: Diptera) do Instituto Nacional de Pesquisas da Amazônia (INPA), Manaus, Amazonas, Brasil. Boletim do Museu Paraense Emílio Goeldi, série Zoologia, 11(1), pp.57-99.

Henriques AL, Ferreira RLM, Vidal JF, Rafael JA. *Betrequia ocellata* Oldroyd (Diptera, Tabanidae, Rhinomyzini) blood feeding on *Caiman crocodilus* (Linnaeus) (Crocodylia, Alligatoridae) in Manaus, Brazil. Revista Brasileira de Zoologia. 2000; 17:609-13.

Henriques, A. L. 2004. Tabanidae (Insecta: Diptera) do Parque Nacional do Jaú II. In: Fundação Vitória Amazônica (FVA). Projeto Janelas para a Biodiversidade. Inventários Biológicos. Cap. 09. pp. 143-152.

Krolow TK, Krüger RF, Ribeiro PB: Chave pictórica para os gêneros de Tabanidae (Insecta: Diptera) do bioma Campos Sulinos, Rio Grande do Sul, Brasil. Biota Neotropica 2007, **7**:253–64.

Krolow TK, Henriques AL, Rafael JA. Tabanidae (Diptera) no dossel da floresta amazônica atraídos por luz e descrição de machos de três espécies. Acta Amazonica. 2010; 40:605-10.

Krolow TK, Henriques AL, Pollet M. The Tabanidae of the Mitaraka expedition, with an update check list of French Guiana. ZooKeys. 2017; 684,85-118. doi: 10.3897/zookeys.684.13197.

Leclercq M. Tabanidae (Diptera) de la région néotropicale. Bulletin Inst Agron. Stat Recherches Gembloux. 1965; 3:376-80.

Lima HIL, Krolow TK, Henriques AL. Checklist of horse flies (Diptera: Tabanidae) from Taquaruçu, Tocantins, Brazil with newrecords for the state. Check List. 2015. doi:http://dx.doi.org/10.15560/11.2.1596.

Limeira de Oliveira, F. 2003. Taxonomia de adultos de mutucas (Diptera: Tabanidae) do estado do Maranhão, Brasil. Programa de Pós-Graduação em Biologia Tropical e Recursos Naturais do Instituto Nacional de Pesquisas da Amazônia (INPA) e Universidade Federal do Amazonas. p. 159-160.

Lutz, A., Neiva, A. 1914. As Tabanidae do Estado do Rio de Janeiro. Memórias do Instituto Oswaldo Cruz, 6 (2), pp. 282-287.

Luz-Alves WC, Gorayeb IS, Silva JCL, Loureiro ECB. Bactérias transportadas em mutucas (Diptera: Tabanidae) no nordeste do estado do Pará, Brasil. Boletim do Museu Paraense Emílio Goeldi – Ciências Naturais. 2007;3:11-20.

Manrique-Saide P, Delfin-Gonzalez H, Ibañez-Bernal S. Horseflies (Diptera: Tabanidae) from protected areas of the Yucatan Peninsula, México. Florida Entomologist. 2001; 84:352-62.

Meijer, A. 2014. Testando um Controlador de Mutucas. Cartas da Mata Atlântica (154). Correio do Litoral, Paraná. p. 1-14.

Oliveira AF, Ferreira RLM, Rafael JA. Sazonalidade e atividade diurna de Tabanidae (Diptera:Insecta) de Dossel na Reserva Florestal Adolpho Ducke, Manaus, AM. Neotropical Entomology. 2007; 36:790-97.

Patrick CR, Hays KL. Some Tabanidae (Diptera) from eastern Ecuador.The Florida Entomologist. 1968; 51:219-21.

Rafael JA, Charlwood JD. Idade fisiológica, variação sazonal e periodicidade diurna de quatro populaçõesde Tabanidae (Diptera) no Campus Universitário, Manaus, Brasil. Acta Amazonica. 1980; 10:907-27.

Rafael, J. A. 1982. Ocorrência sazonal e abundância relativa de Tabanidae (Diptera) no campus universitário, Manaus, Amazonas. Acta Amazônica, 12 (1) 225-229.

Rafael, J.A., Gorayeb, I. S., Rosa, M. S. S., Henriques, A. L. 1991. Tabanidae (Diptera) da Ilha de Maracá e Serra Pacaraima, Roraima, Brasil, com descrição de duas espécies novas. Acta Amazonica, 21 (1), pp. 351-367.

Raymond HL. Action des taons (Diptera, Tabanidae) sur le comportement d’um troupeau de zébus au pâturage en Guyane française. 1987; 36:375-86.

Raymond HL. Distribution temporelle des principales espèces de Taons: (Diptera: Tabanidae) nuisibles au bétail en Guyane Française. Analles de la Société Entomologique de France. 1989; 25:289-94.

Ríos MV, Gómez ZT, Gorayeb IS, Tamasaukas R. Abundancia estacional de tabánidos (Diptera: Tabanidae) en el sector Las Lajas, Municipio Miranda, estado Guárico, Venezuela. Entomotropica. 2004; 19:149-52.

Strickman D. Notes on abanidae (Diptera) from Paraguay. Journal Medical Entological. 1982; 19:399-402.

Turcatel, M., Carbalho, C. J. B., Rafael, J. A. 2007. Mutucas (Diptera: Tabanidae) do estado do Paraná, Brasil: chave de identificação pictórica para subfamílias, tribos e gêneros. Biota Neotropica, 7 (2), pp. 265-278.

Wilkerson RC, Fairchild GB.A checklist and generic key to the Tabanidae (Diptera) of perú with special reference to the Tambopata Reserved Zone, Madre de Dios. Revista Peruana de Entomologia. 1985; 27:37-53.
