# Supplementary material for: Spatial epidemiology of Tabanus (Diptera: Tabanidae) vectors of Trypanosoma
Source: Parasit Vectors. 2025 Apr 3;18:128. doi: 10.1186/s13071-025-06708-z (PMC11969902; doi:10.1186/s13071-025-06708-z)
Supplement: Supplementary file 2 — Supplementary Material 2. List of the occurrence records georeferenced in the Neotropical region of the six Tabanus species used in ecological niche modeling. These occurrences are results after the filter of the 5-km area. The coordinates are represented in decimal degrees. [file 13071_2025_6708_MOESM2_ESM.doc]

Additional file 2. List of the occurrence records georeferenced in the Neotropical region of the six *Tabanus* species used in ecological niche modelling. These occurrences are results after the filter of the 5km. The coordinates are represented in decimal degrees.

| Species | Longitude | Latitude |
| --- | --- | --- |
| *Tabanus claripennis* | -36.8808 | -7.4708 |
| *Tabanus claripennis* | -37.4452 | -7.0388 |
| *Tabanus claripennis* | -42.3000 | -2.9600 |
| *Tabanus claripennis* | -42.7761 | -2.7275 |
| *Tabanus claripennis* | -43.3558 | -4.8588 |
| *Tabanus claripennis* | -43.4147 | -4.8916 |
| *Tabanus claripennis* | -43.8100 | -5.1138 |
| *Tabanus claripennis* | -44.0438 | -6.7338 |
| *Tabanus claripennis* | -44.0769 | -6.6019 |
| *Tabanus claripennis* | -44.6488 | -5.2577 |
| *Tabanus claripennis* | -46.7727 | -1.0627 |
| *Tabanus claripennis* | -47.8827 | -15.7938 |
| *Tabanus claripennis* | -47.9166 | -15.9333 |
| *Tabanus claripennis* | -48.5388 | -12.0250 |
| *Tabanus claripennis* | -48.5391 | -12.0250 |
| *Tabanus claripennis* | -50.9000 | -31.0630 |
| *Tabanus claripennis* | -50.9601 | -31.2130 |
| *Tabanus claripennis* | -50.9605 | -31.2136 |
| *Tabanus claripennis* | -51.0500 | -30.0400 |
| *Tabanus claripennis* | -51.3333 | -6.0000 |
| *Tabanus claripennis* | -51.7923 | -31.1215 |
| *Tabanus claripennis* | -52.0600 | -31.4640 |
| *Tabanus claripennis* | -52.1217 | -31.4329 |
| *Tabanus claripennis* | -52.1395 | -31.5643 |
| *Tabanus claripennis* | -52.1456 | -31.5567 |
| *Tabanus claripennis* | -52.2176 | -31.6718 |
| *Tabanus claripennis* | -52.2227 | -31.7441 |
| *Tabanus claripennis* | -52.2542 | -31.7217 |
| *Tabanus claripennis* | -52.3427 | -31.7719 |
| *Tabanus claripennis* | -52.3427 | -31.7719 |
| *Tabanus claripennis* | -52.3528 | -31.6278 |
| *Tabanus claripennis* | -52.4036 | -31.8044 |
| *Tabanus claripennis* | -52.4865 | -32.6351 |
| *Tabanus claripennis* | -52.5097 | -32.5605 |
| *Tabanus claripennis* | -52.5253 | -32.5334 |
| *Tabanus claripennis* | -52.5371 | -32.5384 |
| *Tabanus claripennis* | -52.5373 | -32.5386 |
| *Tabanus claripennis* | -52.5916 | -31.6727 |
| *Tabanus claripennis* | -53.1991 | -32.2227 |
| *Tabanus claripennis* | -53.7500 | -27.3666 |
| *Tabanus claripennis* | -53.8533 | -27.2325 |
| *Tabanus claripennis* | -54.8288 | -19.8319 |
| *Tabanus claripennis* | -54.8438 | -18.9177 |
| *Tabanus claripennis* | -55.7869 | -20.4708 |
| *Tabanus claripennis* | -56.5911 | -16.3850 |
| *Tabanus claripennis* | -56.6500 | -18.9833 |
| *Tabanus claripennis* | -56.7150 | -20.5386 |
| *Tabanus claripennis* | -57.0666 | -19.2666 |
| *Tabanus claripennis* | -57.4166 | -25.2500 |
| *Tabanus claripennis* | -57.4166 | -25.3333 |
| *Tabanus claripennis* | -57.5300 | 6.0300 |
| *Tabanus claripennis* | -57.6533 | -19.0091 |
| *Tabanus claripennis* | -57.6666 | -25.3333 |
| *Tabanus claripennis* | -57.8825 | -21.6988 |
| *Tabanus claripennis* | -57.8827 | -21.6988 |
| *Tabanus claripennis* | -58.4331 | -34.6120 |
| *Tabanus claripennis* | -58.4500 | -34.6000 |
| *Tabanus claripennis* | -58.6020 | -33.7511 |
| *Tabanus claripennis* | -58.6020 | -33.7511 |
| *Tabanus claripennis* | -58.6578 | -33.7139 |
| *Tabanus claripennis* | -58.8658 | -34.1755 |
| *Tabanus claripennis* | -58.8722 | -33.8330 |
| *Tabanus claripennis* | -58.9166 | -34.1666 |
| *Tabanus claripennis* | -58.9500 | -34.1666 |
| *Tabanus claripennis* | -59.8327 | 3.3600 |
| *Tabanus claripennis* | -59.8333 | 3.6333 |
| *Tabanus claripennis* | -60.1627 | 4.6305 |
| *Tabanus claripennis* | -60.1680 | 4.5961 |
| *Tabanus claripennis* | -60.6714 | 2.8195 |
| *Tabanus claripennis* | -60.6719 | 2.8200 |
| *Tabanus claripennis* | -60.9436 | -2.6258 |
| *Tabanus claripennis* | -60.9436 | -2.0388 |
| *Tabanus claripennis* | -60.9841 | 1.4544 |
| *Tabanus claripennis* | -61.5833 | 3.4166 |
| *Tabanus claripennis* | -61.6669 | 3.4169 |
| *Tabanus claripennis* | -61.7500 | -1.9500 |
| *Tabanus claripennis* | -62.5461 | -2.3944 |
| *Tabanus claripennis* | -62.6666 | -2.2500 |
| *Tabanus claripennis* | -63.1536 | -17.9436 |
| *Tabanus claripennis* | -67.4333 | 8.9333 |
| *Tabanus claripennis* | -67.4397 | 8.9297 |
| *Tabanus claripennis* | -68.1344 | -16.5032 |
| *Tabanus claripennis* | -69.9857 | 11.2187 |
| *Tabanus claripennis* | -70.4018 | 18.9294 |
| *Tabanus claripennis* | -70.6480 | -33.4566 |
| *Tabanus claripennis* | -70.6833 | 19.8000 |
| *Tabanus claripennis* | -70.7222 | -33.1425 |
| *Tabanus claripennis* | -70.7833 | -37.2166 |
| *Tabanus claripennis* | -70.8667 | -33.6666 |
| *Tabanus claripennis* | -70.9333 | -33.6666 |
| *Tabanus claripennis* | -70.9833 | 19.5883 |
| *Tabanus claripennis* | -71.0136 | -32.9755 |
| *Tabanus claripennis* | -71.1666 | -34.4000 |
| *Tabanus claripennis* | -71.1700 | 19.2800 |
| *Tabanus claripennis* | -71.4347 | -34.2747 |
| *Tabanus claripennis* | -71.6074 | -33.5947 |
| *Tabanus claripennis* | -72.2500 | -38.2500 |
| *Tabanus claripennis* | -72.7166 | -37.8000 |
| *Tabanus claripennis* | -72.7880 | -45.5050 |
| *Tabanus claripennis* | -73.0000 | 3.5000 |
| *Tabanus claripennis* | -73.0500 | -36.8333 |
| *Tabanus claripennis* | -73.2516 | -1.7834 |
| *Tabanus claripennis* | -74.0690 | 11.2510 |
| *Tabanus claripennis* | -74.1420 | 11.2180 |
| *Tabanus claripennis* | -75.1666 | 7.9500 |
| *Tabanus claripennis* | -75.1833 | 8.0000 |
| *Tabanus claripennis* | -76.3333 | 3.4166 |
| *Tabanus claripennis* | -76.5222 | 3.4566 |
| *Tabanus claripennis* | -76.5224 | 3.4566 |
| *Tabanus claripennis* | -78.2700 | 22.4000 |
| *Tabanus claripennis* | -85.2100 | 9.9000 |
| *Tabanus claripennis* | -85.2966 | 9.8789 |
| *Tabanus claripennis* | -85.3514 | 10.3504 |
| *Tabanus claripennis* | -85.3523 | 10.3491 |
| *Tabanus claripennis* | -85.3523 | 10.3491 |
| *Tabanus claripennis* | -85.3832 | 10.3666 |
| *Tabanus claripennis* | -85.3832 | 10.3666 |
| **Total occurrences:** | **117** |  |
|  |  |  |
| Species | Longitude | Latitude |
| *Tabanus importunus* | -35.2000 | -5.8391 |
| *Tabanus importunus* | -35.2166 | -5.7833 |
| *Tabanus importunus* | -42.2570 | -2.949 |
| *Tabanus importunus* | -42.3933 | -3.3108 |
| *Tabanus importunus* | -42.4188 | -3.3611 |
| *Tabanus importunus* | -42.7761 | -2.7275 |
| *Tabanus importunus* | -43.2075 | -22.9027 |
| *Tabanus importunus* | -43.3558 | -4.8588 |
| *Tabanus importunus* | -43.4147 | -4.8916 |
| *Tabanus importunus* | -43.8100 | -5.1138 |
| *Tabanus importunus* | -44.6100 | -2.2340 |
| *Tabanus importunus* | -44.6488 | -5.2577 |
| *Tabanus importunus* | -44.7833 | -4.2333 |
| *Tabanus importunus* | -45.0594 | -3.1641 |
| *Tabanus importunus* | -45.3427 | -2.2008 |
| *Tabanus importunus* | -46.7727 | -1.0627 |
| *Tabanus importunus* | -46.7727 | -1.3833 |
| *Tabanus importunus* | -46.9377 | -12.9308 |
| *Tabanus importunus* | -46.9383 | -12.9313 |
| *Tabanus importunus* | -47.1144 | -7.4586 |
| *Tabanus importunus* | -47.1666 | -0.7666 |
| *Tabanus importunus* | -47.4033 | -7.0744 |
| *Tabanus importunus* | -47.4427 | -5.5816 |
| *Tabanus importunus* | -47.4688 | -7.3327 |
| *Tabanus importunus* | -47.4833 | -5.7833 |
| *Tabanus importunus* | -47.6300 | -0.8620 |
| *Tabanus claripennis* | -47.9166 | -15.9333 |
| *Tabanus importunus* | -48.1250 | -10.2497 |
| *Tabanus importunus* | -48.1252 | -10.2666 |
| *Tabanus importunus* | -48.1260 | -10.2506 |
| *Tabanus importunus* | -48.1265 | -10.2491 |
| *Tabanus importunus* | -48.1580 | -5.1727 |
| *Tabanus importunus* | -48.2336 | -11.9750 |
| *Tabanus importunus* | -52.2675 | 1.6552 |
| *Tabanus importunus* | -48.2450 | -1.3608 |
| *Tabanus importunus* | -48.2938 | -1.2238 |
| *Tabanus importunus* | -48.4169 | -10.7077 |
| *Tabanus importunus* | -48.4172 | -10.7080 |
| *Tabanus importunus* | -48.4500 | -1.4686 |
| *Tabanus importunus* | -48.5110 | -1.6020 |
| *Tabanus importunus* | -48.5388 | -12.0250 |
| *Tabanus importunus* | -48.5391 | -12.0250 |
| *Tabanus importunus* | -48.9666 | -1.0166 |
| *Tabanus importunus* | -49.6538 | -10.7519 |
| *Tabanus importunus* | -49.7000 | -3.7000 |
| *Tabanus importunus* | -50.0377 | -9.9783 |
| *Tabanus importunus* | -50.0379 | -9.9784 |
| *Tabanus importunus* | -50.0802 | -6.0677 |
| *Tabanus importunus* | -50.1090 | 0.0940 |
| *Tabanus importunus* | -50.8000 | -2.0500 |
| *Tabanus importunus* | -51.0500 | 0.0333 |
| *Tabanus importunus* | -51.2890 | -0.0800 |
| *Tabanus importunus* | -51.3333 | -6.0000 |
| *Tabanus importunus* | -51.4000 | 0.7166 |
| *Tabanus importunus* | -52.1666 | 4.5500 |
| *Tabanus importunus* | -52.3666 | -1.0666 |
| *Tabanus importunus* | -52.9515 | 5.3750 |
| *Tabanus importunus* | -54.0666 | -2.0166 |
| *Tabanus importunus* | -54.2666 | -2.2500 |
| *Tabanus importunus* | -54.7077 | -2.4427 |
| *Tabanus importunus* | -54.8288 | -19.8319 |
| *Tabanus importunus* | -54.8294 | -19.8316 |
| *Tabanus importunus* | -54.8438 | -18.9177 |
| *Tabanus importunus* | -54.8441 | -18.9180 |
| *Tabanus importunus* | -54.9544 | -2.5047 |
| *Tabanus importunus* | -55.7497 | -15.4605 |
| *Tabanus importunus* | -55.7500 | -15.4608 |
| *Tabanus importunus* | -55.7869 | -20.4708 |
| *Tabanus importunus* | -55.7872 | -20.4711 |
| *Tabanus importunus* | -55.9838 | -4.2758 |
| *Tabanus importunus* | -56.2227 | -16.2566 |
| *Tabanus importunus* | -56.5027 | -23.4405 |
| *Tabanus importunus* | -56.5833 | -1.3333 |
| *Tabanus importunus* | -56.6227 | -16.2569 |
| *Tabanus importunus* | -56.6500 | -18.9833 |
| *Tabanus importunus* | -56.6680 | -22.9194 |
| *Tabanus importunus* | -56.7150 | -20.5388 |
| *Tabanus importunus* | -57.0666 | -19.2666 |
| *Tabanus importunus* | -57.0833 | -24.1000 |
| *Tabanus importunus* | -57.1333 | -24.1333 |
| *Tabanus importunus* | -57.6533 | -19.0091 |
| *Tabanus importunus* | -57.8825 | -21.6988 |
| *Tabanus importunus* | -58.3421 | -15.8113 |
| *Tabanus importunus* | -58.3833 | -1.0833 |
| *Tabanus importunus* | -58.6322 | -26.2416 |
| *Tabanus importunus* | -58.8402 | -27.4711 |
| *Tabanus importunus* | -59.4500 | -10.1666 |
| *Tabanus importunus* | -59.4666 | -14.4500 |
| *Tabanus importunus* | -59.5932 | -18.3381 |
| *Tabanus importunus* | -59.6000 | 4.0500 |
| *Tabanus importunus* | -59.6227 | 3.8808 |
| *Tabanus importunus* | -59.8327 | 3.3544 |
| *Tabanus importunus* | -59.8330 | 3.3555 |
| *Tabanus importunus* | -59.8333 | 3.6333 |
| *Tabanus importunus* | -59.8619 | 3.3933 |
| *Tabanus importunus* | -59.9308 | -2.8630 |
| *Tabanus importunus* | -59.9508 | -15.0077 |
| *Tabanus importunus* | -59.9666 | -14.8166 |
| *Tabanus importunus* | -59.9677 | -3.0944 |
| *Tabanus importunus* | -60.0094 | -3.1352 |
| *Tabanus importunus* | -60.0166 | -3.1333 |
| *Tabanus importunus* | -60.1166 | -12.7166 |
| *Tabanus importunus* | -60.1188 | -5.2647 |
| *Tabanus importunus* | -60.1458 | -12.7405 |
| *Tabanus importunus* | -60.1627 | 4.6305 |
| *Tabanus importunus* | -60.3355 | -5.1522 |
| *Tabanus importunus* | -60.3905 | -5.2177 |
| *Tabanus importunus* | -60.6719 | 2.8200 |
| *Tabanus importunus* | -60.9841 | 1.4544 |
| *Tabanus importunus* | -60.9936 | -1.4544 |
| *Tabanus importunus* | -61.0030 | 1.4897 |
| *Tabanus importunus* | -61.1166 | 4.4500 |
| *Tabanus importunus* | -61.1458 | 4.4308 |
| *Tabanus importunus* | -61.1477 | 4.4813 |
| *Tabanus importunus* | -61.4344 | 3.3652 |
| *Tabanus importunus* | -61.4452 | 3.3591 |
| *Tabanus importunus* | -61.5897 | -1.9127 |
| *Tabanus importunus* | -61.6666 | -3.1416 |
| *Tabanus importunus* | -61.7277 | 3.7458 |
| *Tabanus importunus* | -61.7333 | -1.8833 |
| *Tabanus importunus* | -61.7352 | -1.8877 |
| *Tabanus importunus* | -61.8166 | -1.9500 |
| *Tabanus importunus* | -61.9935 | -1.4544 |
| *Tabanus importunus* | -62.3288 | 1.4911 |
| *Tabanus importunus* | -62.6666 | -2.2500 |
| *Tabanus importunus* | -62.7833 | -14.7333 |
| *Tabanus importunus* | -62.8952 | -10.3391 |
| *Tabanus importunus* | -63.2833 | 2.2333 |
| *Tabanus importunus* | -63.3252 | -10.2944 |
| *Tabanus importunus* | -63.6122 | -10.5969 |
| *Tabanus importunus* | -63.8743 | -8.7372 |
| *Tabanus importunus* | -63.9038 | -8.7619 |
| *Tabanus importunus* | -65.0833 | -10.9666 |
| *Tabanus importunus* | -65.0941 | -10.9730 |
| *Tabanus importunus* | -66.6194 | -14.1724 |
| *Tabanus importunus* | -67.4397 | 8.9297 |
| *Tabanus importunus* | -69.9327 | 4.2052 |
| *Tabanus importunus* | -69.9857 | 11.2187 |
| *Tabanus importunus* | -72.6136 | 3.6952 |
| *Tabanus importunus* | -73.3931 | 4.1415 |
| *Tabanus importunus* | -73.6265 | 4.1504 |
| *Tabanus importunus* | -73.8629 | -12.5072 |
| *Tabanus importunus* | -74.1037 | 5.5336 |
| *Tabanus importunus* | -74.1875 | 10.5980 |
| *Tabanus importunus* | -75.1833 | 8.0000 |
| *Tabanus importunus* | -80.5000 | 8.4000 |
| *Tabanus importunus* | -85.3523 | 10.3491 |
| *Tabanus importunus* | -85.3736 | 10.1682 |
| *Tabanus importunus* | -85.4952 | 10.9625 |
| **Total occurrences:** | **149** |  |
|  |  |  |
| Species | Longitude | Latitude |
| *Tabanus nebulosus* | -46.7727 | -1.0627 |
| *Tabanus nebulosus* | -47.3000 | -1.2000 |
| *Tabanus nebulosus* | -47.4758 | -5.5258 |
| *Tabanus nebulosus* | -48.4790 | -1.456 |
| *Tabanus nebulosus* | -50.1640 | 0.1633 |
| *Tabanus nebulosus* | -50.3166 | -27.8000 |
| *Tabanus nebulosus* | -50.3425 | -9.3308 |
| *Tabanus nebulosus* | -51.0500 | 0.0333 |
| *Tabanus nebulosus* | -51.1727 | -0.0455 |
| *Tabanus nebulosus* | -51.2888 | -0.1150 |
| *Tabanus nebulosus* | -51.3333 | -6.0000 |
| *Tabanus nebulosus* | -56.6500 | -18.9833 |
| *Tabanus nebulosus* | -57.1333 | -24.1333 |
| *Tabanus nebulosus* | -57.5284 | -27.5502 |
| *Tabanus nebulosus* | -57.7211 | -25.2898 |
| *Tabanus nebulosus* | -58.1815 | -26.1725 |
| *Tabanus nebulosus* | -58.2376 | -18.9714 |
| *Tabanus nebulosus* | -58.9333 | -27.3331 |
| *Tabanus nebulosus* | -59.0306 | -27.4188 |
| *Tabanus nebulosus* | -59.4666 | -14.4500 |
| *Tabanus nebulosus* | -59.5932 | -18.3381 |
| *Tabanus nebulosus* | -59.9666 | -14.8166 |
| *Tabanus nebulosus* | -60.7014 | -31.6191 |
| *Tabanus nebulosus* | -61.0056 | 8.5821 |
| *Tabanus nebulosus* | -61.5833 | 3.4166 |
| *Tabanus nebulosus* | -62.2158 | -10.7480 |
| *Tabanus nebulosus* | -64.3494 | -30.4243 |
| *Tabanus nebulosus* | -64.5500 | -10.3166 |
| *Tabanus nebulosus* | -65.0833 | -10.9666 |
| *Tabanus nebulosus* | -65.2150 | -26.8287 |
| *Tabanus nebulosus* | -67.4397 | 8.9297 |
| *Tabanus nebulosus* | -67.5327 | -9.5877 |
| *Tabanus nebulosus* | -67.5551 | -39.0263 |
| *Tabanus nebulosus* | -69.9857 | 11.2187 |
| *Tabanus nebulosus* | -73.3931 | 4.1415 |
| *Tabanus nebulosus* | -74.0520 | 11.081 |
| *Tabanus nebulosus* | -74.1583 | 10.7730 |
| *Tabanus nebulosus* | -74.7969 | -10.9641 |
| *Tabanus nebulosus* | -75.2166 | 7.9333 |
| *Tabanus nebulosus* | -75.5280 | 6.2438 |
| *Tabanus nebulosus* | -76.3000 | 3.9000 |
| *Tabanus nebulosus* | -77.7275 | 8.1326 |
| *Tabanus nebulosus* | -79.8585 | 8.7591 |
| *Tabanus nebulosus* | -80.5000 | 8.4000 |
| *Tabanus nebulosus* | -84.1237 | 9.3877 |
| *Tabanus nebulosus* | -84.6081 | 9.7742 |
| *Tabanus nebulosus* | -84.7888 | 10.8938 |
| *Tabanus nebulosus* | -85.2623 | 10.3326 |
| *Tabanus nebulosus* | -85.3173 | 10.2533 |
| *Tabanus nebulosus* | -85.3508 | 10.1700 |
| *Tabanus nebulosus* | -85.3514 | 10.3504 |
| *Tabanus nebulosus* | -85.3523 | 10.3491 |
| *Tabanus nebulosus* | -85.3571 | 10.1519 |
| *Tabanus nebulosus* | -85.3585 | 10.1538 |
| *Tabanus nebulosus* | -85.3713 | 10.1614 |
| *Tabanus nebulosus* | -85.3736 | 10.1682 |
| *Tabanus nebulosus* | -85.3791 | 10.1698 |
| *Tabanus nebulosus* | -85.5735 | 10.8655 |
| *Tabanus nebulosus* | -85.5996 | 10.1709 |
| *Tabanus nebulosus* | -85.6154 | 10.8364 |
| **Total occurrences:** | **60** |  |
|  |  |  |
| Species | Longitude | Latitude |
| *Tabanus pungens* | -46.7727 | -1.0627 |
| *Tabanus pungens* | -47.3000 | -1.2000 |
| *Tabanus pungens* | -47.6350 | -24.3208 |
| *Tabanus pungens* | -48.2450 | -1.3608 |
| *Tabanus pungens* | -48.2938 | -1.2238 |
| *Tabanus pungens* | -48.4891 | -1.4558 |
| *Tabanus pungens* | -48.5810 | -25.806 |
| *Tabanus pungens* | -48.8166 | -25.4666 |
| *Tabanus pungens* | -49.2650 | -8.2577 |
| *Tabanus pungens* | -49.5666 | -0.9666 |
| *Tabanus pungens* | -49.7000 | -3.7000 |
| *Tabanus pungens* | -50.1667 | 0.16456 |
| *Tabanus pungens* | -50.9605 | -31.2136 |
| *Tabanus pungens* | -51.0500 | 0.0333 |
| *Tabanus pungens* | -51.2888 | -0.1150 |
| *Tabanus pungens* | -51.3333 | 0.1666 |
| *Tabanus pungens* | -52.2227 | -31.7441 |
| *Tabanus pungens* | -52.4036 | -31.8044 |
| *Tabanus pungens* | -52.5916 | -31.6727 |
| *Tabanus pungens* | -53.1991 | -32.2227 |
| *Tabanus pungens* | -54.9500 | -34.9000 |
| *Tabanus pungens* | -56.4414 | -25.7764 |
| *Tabanus pungens* | -56.6500 | -18.9833 |
| *Tabanus pungens* | -57.1000 | -1.5333 |
| *Tabanus pungens* | -57.4166 | -25.3333 |
| *Tabanus pungens* | -58.1456 | 6.7991 |
| *Tabanus pungens* | -58.6020 | -33.7511 |
| *Tabanus pungens* | -58.8722 | -33.8330 |
| *Tabanus pungens* | -59.9632 | -3.0893 |
| *Tabanus pungens* | -59.9666 | -3.2500 |
| *Tabanus pungens* | -60.0166 | -3.1000 |
| *Tabanus pungens* | -60.6773 | -16.7868 |
| *Tabanus pungens* | -61.3666 | -1.9333 |
| *Tabanus pungens* | -62.6666 | -2.2500 |
| *Tabanus pungens* | -63.0998 | 10.1144 |
| *Tabanus pungens* | -63.1536 | -17.9436 |
| *Tabanus pungens* | -63.7810 | -18.8306 |
| *Tabanus pungens* | -65.0833 | -10.9666 |
| *Tabanus pungens* | -66.6194 | -14.1724 |
| *Tabanus pungens* | -67.3500 | 9.9166 |
| *Tabanus pungens* | -67.4136 | 9.8695 |
| *Tabanus pungens* | -67.4397 | 8.9297 |
| *Tabanus pungens* | -68.8268 | -32.9036 |
| *Tabanus pungens* | -69.9857 | 11.2187 |
| *Tabanus pungens* | -72.6000 | -7.6333 |
| *Tabanus pungens* | -73.3931 | 4.1415 |
| *Tabanus pungens* | -73.6265 | 4.1504 |
| *Tabanus pungens* | -76.1672 | 4.2102 |
| *Tabanus pungens* | -77.0450 | -12.0457 |
| *Tabanus pungens* | -77.7316 | -1.0319 |
| *Tabanus pungens* | -78.0280 | -2.1460 |
| *Tabanus pungens* | -78.0921 | -1.4779 |
| *Tabanus pungens* | -78.5695 | -9.0767 |
| *Tabanus pungens* | -79.1666 | -2.2222 |
| *Tabanus pungens* | -79.1952 | -2.2036 |
| *Tabanus pungens* | -79.2209 | -7.8898 |
| *Tabanus pungens* | -79.2944 | -2.4638 |
| *Tabanus pungens* | -79.3361 | -2.4222 |
| *Tabanus pungens* | -79.3875 | -1.7102 |
| *Tabanus pungens* | -79.3884 | -2.2305 |
| *Tabanus pungens* | -79.4497 | -3.9494 |
| *Tabanus pungens* | -79.4602 | -1.0316 |
| *Tabanus pungens* | -79.4666 | 0.3330 |
| *Tabanus pungens* | -79.5883 | -2.1313 |
| *Tabanus pungens* | -79.6944 | -2.7383 |
| *Tabanus pungens* | -79.7230 | -1.9588 |
| *Tabanus pungens* | -79.8125 | -2.9066 |
| *Tabanus pungens* | -79.8847 | 0.2150 |
| *Tabanus pungens* | -79.8944 | -2.1958 |
| *Tabanus pungens* | -79.9752 | -2.1561 |
| *Tabanus pungens* | -79.9863 | -1.7130 |
| *Tabanus pungens* | -80.0086 | -1.9077 |
| *Tabanus pungens* | -80.1279 | 8.6201 |
| *Tabanus pungens* | -80.2669 | -2.3300 |
| *Tabanus pungens* | -80.5000 | 8.4000 |
| *Tabanus pungens* | -80.6240 | -1.4755 |
| *Tabanus pungens* | -80.6675 | -2.0175 |
| *Tabanus pungens* | -80.7155 | -1.8333 |
| *Tabanus pungens* | -82.9562 | 9.5193 |
| *Tabanus pungens* | -83.2831 | 8.7593 |
| *Tabanus pungens* | -83.5667 | 8.6790 |
| *Tabanus pungens* | -83.7165 | 10.5942 |
| *Tabanus pungens* | -84.0109 | 10.4391 |
| *Tabanus pungens* | -84.7888 | 10.8938 |
| *Tabanus pungens* | -85.3523 | 10.3491 |
| *Tabanus pungens* | -85.3736 | 10.1682 |
| *Tabanus pungens* | -85.3819 | 9.8508 |
| *Tabanus pungens* | -85.5769 | 11.0336 |
| *Tabanus pungens* | -88.3333 | 21.4067 |
| *Tabanus pungens* | -88.8411 | 15.4723 |
| *Tabanus pungens* | -89.6441 | 15.4002 |
| *Tabanus pungens* | -90.4134 | 20.7969 |
| *Tabanus pungens* | -92.9076 | 18.1228 |
| **Total occurrences:** | **93** |  |
|  |  |  |
| Species | Longitude | Latitude |
| *Tabanus sorbillans* | -42.3933 | -3.3108 |
| *Tabanus sorbillans* | -42.4177 | -3.3613 |
| *Tabanus sorbillans* | -42.5180 | -3.2816 |
| *Tabanus sorbillans* | -43.3344 | -4.8722 |
| *Tabanus sorbillans* | -43.3561 | -4.8588 |
| *Tabanus sorbillans* | -44.4900 | -5.2900 |
| *Tabanus sorbillans* | -44.6208 | -4.5719 |
| *Tabanus sorbillans* | -44.6277 | -4.5377 |
| *Tabanus sorbillans* | -44.6488 | -5.2577 |
| *Tabanus sorbillans* | -44.7833 | -3.9500 |
| *Tabanus sorbillans* | -45.3425 | -2.2008 |
| *Tabanus sorbillans* | -45.3483 | -3.6813 |
| *Tabanus sorbillans* | -45.3800 | -3.6666 |
| *Tabanus sorbillans* | -45.6652 | -2.9527 |
| *Tabanus sorbillans* | -45.8975 | -3.0455 |
| *Tabanus sorbillans* | -46.2083 | -1.8011 |
| *Tabanus sorbillans* | -46.3033 | -1.7875 |
| *Tabanus sorbillans* | -46.7727 | -1.0627 |
| *Tabanus sorbillans* | -47.1144 | -7.4586 |
| *Tabanus sorbillans* | -47.2500 | -7.3983 |
| *Tabanus sorbillans* | -47.3000 | -1.2000 |
| *Tabanus sorbillans* | -47.5000 | -7.3500 |
| *Tabanus sorbillans* | -48.0166 | -1.5166 |
| *Tabanus sorbillans* | -48.2000 | -7.2000 |
| *Tabanus sorbillans* | -48.2450 | -1.3608 |
| *Tabanus sorbillans* | -48.2938 | -1.2238 |
| *Tabanus sorbillans* | -48.4500 | -1.4666 |
| *Tabanus sorbillans* | -48.4558 | -25.2233 |
| *Tabanus sorbillans* | -48.4900 | -1.455 |
| *Tabanus sorbillans* | -48.5044 | -1.4558 |
| *Tabanus sorbillans* | -48.6597 | -25.3180 |
| *Tabanus sorbillans* | -48.6630 | -1.5310 |
| *Tabanus sorbillans* | -48.8666 | -1.3833 |
| *Tabanus sorbillans* | -48.9527 | -16.3269 |
| *Tabanus sorbillans* | -49.7621 | -29.3797 |
| *Tabanus sorbillans* | -50.1666 | 0.1666 |
| *Tabanus sorbillans* | -51.0500 | 0.0333 |
| *Tabanus sorbillans* | -51.2970 | -30.4730 |
| *Tabanus sorbillans* | -51.9070 | -30.2650 |
| *Tabanus sorbillans* | -52.2000 | 4.5500 |
| *Tabanus sorbillans* | -52.2227 | -31.7441 |
| *Tabanus sorbillans* | -54.2666 | -2.2500 |
| *Tabanus sorbillans* | -54.7077 | -2.4427 |
| *Tabanus sorbillans* | -54.7083 | -2.4430 |
| *Tabanus sorbillans* | -54.8288 | -19.8319 |
| *Tabanus sorbillans* | -54.8438 | -18.9177 |
| *Tabanus sorbillans* | -55.0397 | -1.8461 |
| *Tabanus sorbillans* | -55.7500 | -15.4608 |
| *Tabanus sorbillans* | -55.7869 | -20.4708 |
| *Tabanus sorbillans* | -56.0456 | -28.5515 |
| *Tabanus sorbillans* | -56.6227 | -16.2569 |
| *Tabanus sorbillans* | -56.6500 | -18.9833 |
| *Tabanus sorbillans* | -56.7150 | -20.5388 |
| *Tabanus sorbillans* | -57.0666 | -19.2666 |
| *Tabanus sorbillans* | -57.4166 | -25.3333 |
| *Tabanus sorbillans* | -57.6533 | -19.0091 |
| *Tabanus sorbillans* | -57.8827 | -21.6988 |
| *Tabanus sorbillans* | -58.4160 | -34.1060 |
| *Tabanus sorbillans* | -58.5709 | -34.4483 |
| *Tabanus sorbillans* | -58.7500 | -3.3666 |
| *Tabanus sorbillans* | -58.8001 | -27.4881 |
| *Tabanus sorbillans* | -58.9255 | -34.2310 |
| *Tabanus sorbillans* | -58.9333 | -27.3331 |
| *Tabanus sorbillans* | -58.9705 | -27.2607 |
| *Tabanus sorbillans* | -59.4532 | -28.2439 |
| *Tabanus sorbillans* | -59.5714 | -25.4074 |
| *Tabanus sorbillans* | -59.6105 | -28.4859 |
| *Tabanus sorbillans* | -59.8333 | -2.3333 |
| *Tabanus sorbillans* | -59.8541 | -2.4338 |
| *Tabanus sorbillans* | -59.9308 | -2.8630 |
| *Tabanus sorbillans* | -59.9632 | -3.0893 |
| *Tabanus sorbillans* | -59.9677 | -3.0944 |
| *Tabanus sorbillans* | -60.0058 | -2.9844 |
| *Tabanus sorbillans* | -60.0166 | -3.1333 |
| *Tabanus sorbillans* | -60.0333 | -3.1333 |
| *Tabanus sorbillans* | -60.3797 | -5.1205 |
| *Tabanus sorbillans* | -60.3905 | -5.2177 |
| *Tabanus sorbillans* | -60.6906 | -32.9577 |
| *Tabanus sorbillans* | -60.9657 | -16.3721 |
| *Tabanus sorbillans* | -61.2530 | 10.4580 |
| *Tabanus sorbillans* | -61.3505 | -3.9505 |
| *Tabanus sorbillans* | -61.9419 | -10.8808 |
| *Tabanus sorbillans* | -61.9500 | -10.8666 |
| *Tabanus sorbillans* | -62.0469 | -11.1200 |
| *Tabanus sorbillans* | -62.2158 | -10.7480 |
| *Tabanus sorbillans* | -62.2158 | -10.7480 |
| *Tabanus sorbillans* | -62.2255 | -10.7613 |
| *Tabanus sorbillans* | -62.5000 | -16.6333 |
| *Tabanus sorbillans* | -62.8952 | -10.3391 |
| *Tabanus sorbillans* | -63.0500 | -17.8445 |
| *Tabanus sorbillans* | -63.1536 | -17.9436 |
| *Tabanus sorbillans* | -63.3252 | -10.2944 |
| *Tabanus sorbillans* | -63.6122 | -10.5969 |
| *Tabanus sorbillans* | -63.9000 | -8.7666 |
| *Tabanus sorbillans* | -63.9038 | -8.7619 |
| *Tabanus sorbillans* | -63.9501 | -22.1832 |
| *Tabanus sorbillans* | -64.8669 | -23.4830 |
| *Tabanus sorbillans* | -66.0688 | -2.5408 |
| *Tabanus sorbillans* | -67.1697 | -5.0752 |
| *Tabanus sorbillans* | -67.4397 | 8.9297 |
| *Tabanus sorbillans* | -67.5327 | -3.5877 |
| *Tabanus sorbillans* | -67.7777 | -9.9177 |
| *Tabanus sorbillans* | -67.8100 | -9.9916 |
| *Tabanus sorbillans* | -67.8100 | -9.9747 |
| *Tabanus sorbillans* | -68.9236 | -3.4805 |
| *Tabanus sorbillans* | -69.3416 | -4.3430 |
| *Tabanus sorbillans* | -69.3450 | -4.2555 |
| *Tabanus sorbillans* | -69.8666 | -4.4000 |
| *Tabanus sorbillans* | -69.9088 | -4.2041 |
| *Tabanus sorbillans* | -69.9380 | -4.2525 |
| *Tabanus sorbillans* | -69.9857 | 11.2187 |
| *Tabanus sorbillans* | -70.0344 | -4.3616 |
| *Tabanus sorbillans* | -70.7464 | -13.2285 |
| *Tabanus sorbillans* | -72.6000 | -7.6333 |
| *Tabanus sorbillans* | -72.6700 | -7.6308 |
| *Tabanus sorbillans* | -72.7708 | -7.6169 |
| *Tabanus sorbillans* | -72.7708 | -7.6172 |
| *Tabanus sorbillans* | -73.3931 | 4.1415 |
| *Tabanus sorbillans* | -73.6083 | -7.4505 |
| *Tabanus sorbillans* | -74.6368 | -11.2520 |
| *Tabanus sorbillans* | -76.0000 | -9.2983 |
| *Tabanus sorbillans* | -76.6122 | -0.3966 |
| *Tabanus sorbillans* | -70.7464 | -13.2285 |
| *Tabanus sorbillans* | -72.6000 | -7.6333 |
| *Tabanus sorbillans* | -72.6700 | -7.6308 |
| *Tabanus sorbillans* | -72.7708 | -7.6169 |
| *Tabanus sorbillans* | -72.7708 | -7.6172 |
| *Tabanus sorbillans* | -73.3931 | 4.1415 |
| *Tabanus sorbillans* | -73.6083 | -7.4505 |
| *Tabanus sorbillans* | -74.6368 | -11.2520 |
| *Tabanus sorbillans* | -76.0000 | -9.2983 |
| *Tabanus sorbillans* | -76.6122 | -0.3966 |
| **Total occurrences:** | **132** |  |
|  |  |  |
| Species | Longitude | Latitude |
| *Tabanus triangulum* | -43.1788 | -22.5050 |
| *Tabanus triangulum* | -43.3141 | -22.5787 |
| *Tabanus triangulum* | -43.7930 | -22.9600 |
| *Tabanus triangulum* | -44.5591 | -22.4913 |
| *Tabanus triangulum* | -48.3299 | -25.3098 |
| *Tabanus triangulum* | -48.3643 | -25.5780 |
| *Tabanus triangulum* | -48.3820 | -25.5150 |
| *Tabanus triangulum* | -48.3830 | -25.4710 |
| *Tabanus triangulum* | -48.5088 | -25.5200 |
| *Tabanus triangulum* | -48.5376 | -25.8323 |
| *Tabanus triangulum* | -48.5750 | -25.8827 |
| *Tabanus triangulum* | -48.7430 | -27.0960 |
| *Tabanus triangulum* | -50.8730 | -31.0050 |
| *Tabanus triangulum* | -50.8870 | -31.0042 |
| *Tabanus triangulum* | -50.9601 | -31.2137 |
| *Tabanus triangulum* | -51.1150 | -30.0880 |
| *Tabanus triangulum* | -51.2183 | -30.4132 |
| *Tabanus triangulum* | -51.2190 | -30.3730 |
| *Tabanus triangulum* | -51.2370 | -30.4313 |
| *Tabanus triangulum* | -51.7923 | -31.1215 |
| *Tabanus triangulum* | -51.9490 | -30.2620 |
| *Tabanus triangulum* | -52.0083 | -31.4967 |
| *Tabanus triangulum* | -52.1217 | -31.4329 |
| *Tabanus triangulum* | -52.1395 | -31.5643 |
| *Tabanus triangulum* | -52.1456 | -31.5567 |
| *Tabanus triangulum* | -52.2176 | -31.6711 |
| *Tabanus triangulum* | -52.2542 | -31.7217 |
| *Tabanus triangulum* | -52.3427 | -31.7719 |
| *Tabanus triangulum* | -52.3528 | -31.6278 |
| *Tabanus triangulum* | -52.4036 | -31.8044 |
| *Tabanus triangulum* | -52.4500 | -23.7500 |
| *Tabanus triangulum* | -52.4861 | -32.6339 |
| *Tabanus triangulum* | -52.4865 | -32.6351 |
| *Tabanus triangulum* | -52.5006 | -32.5556 |
| *Tabanus triangulum* | -52.5097 | -32.5605 |
| *Tabanus triangulum* | -52.5253 | -32.5334 |
| *Tabanus triangulum* | -52.5371 | -32.5384 |
| *Tabanus triangulum* | -52.5373 | -32.5386 |
| *Tabanus triangulum* | -52.5576 | -32.5681 |
| *Tabanus triangulum* | -52.5916 | -31.6727 |
| *Tabanus triangulum* | -53.1991 | -32.2227 |
| *Tabanus triangulum* | -54.7506 | -26.5977 |
| *Tabanus triangulum* | -54.9784 | -27.2031 |
| *Tabanus triangulum* | -55.6666 | -27.0000 |
| *Tabanus triangulum* | -56.0443 | -28.5515 |
| *Tabanus triangulum* | -56.1880 | -34.9030 |
| *Tabanus triangulum* | -57.0833 | -25.3333 |
| *Tabanus triangulum* | -57.1333 | -24.1333 |
| *Tabanus triangulum* | -57.1585 | -28.4912 |
| *Tabanus triangulum* | -57.4166 | -25.3333 |
| *Tabanus triangulum* | -57.4166 | -25.2500 |
| *Tabanus triangulum* | -57.5000 | -25.3333 |
| *Tabanus triangulum* | -57.5281 | -27.5494 |
| *Tabanus triangulum* | -57.6666 | -25.3333 |
| *Tabanus triangulum* | -58.0833 | -31.3833 |
| *Tabanus triangulum* | -58.3256 | -31.8636 |
| *Tabanus triangulum* | -58.4500 | -34.6000 |
| *Tabanus triangulum* | -58.6020 | -33.7511 |
| *Tabanus triangulum* | -58.7664 | -27.7032 |
| *Tabanus triangulum* | -58.7873 | -27.4999 |
| *Tabanus triangulum* | -58.8722 | -33.8330 |
| *Tabanus triangulum* | -59.0000 | -34.0000 |
| *Tabanus triangulum* | -61.2820 | -38.9180 |
| *Tabanus triangulum* | -62.9258 | -23.2412 |
| *Tabanus triangulum* | -63.7810 | -18.8306 |
| *Tabanus triangulum* | -63.7981 | -22.5191 |
| *Tabanus triangulum* | -64.4506 | -22.8828 |
| *Tabanus triangulum* | -64.9230 | -24.5839 |
| *Tabanus triangulum* | -65.3275 | -26.7824 |
| *Tabanus triangulum* | -65.5000 | -27.0000 |
| *Tabanus triangulum* | -67.0156 | -24.3243 |
| **Total occurrences:** | **71** |  |
