# Supplementary material for: Spatial epidemiology of Tabanus (Diptera: Tabanidae) vectors of Trypanosoma
Source: Parasit Vectors. 2025 Apr 3;18:128. doi: 10.1186/s13071-025-06708-z (PMC11969902; doi:10.1186/s13071-025-06708-z)

Supplementary file 3a. *Tabanus* species occurrences in the Neotropical region and major rivers in South and Central America.


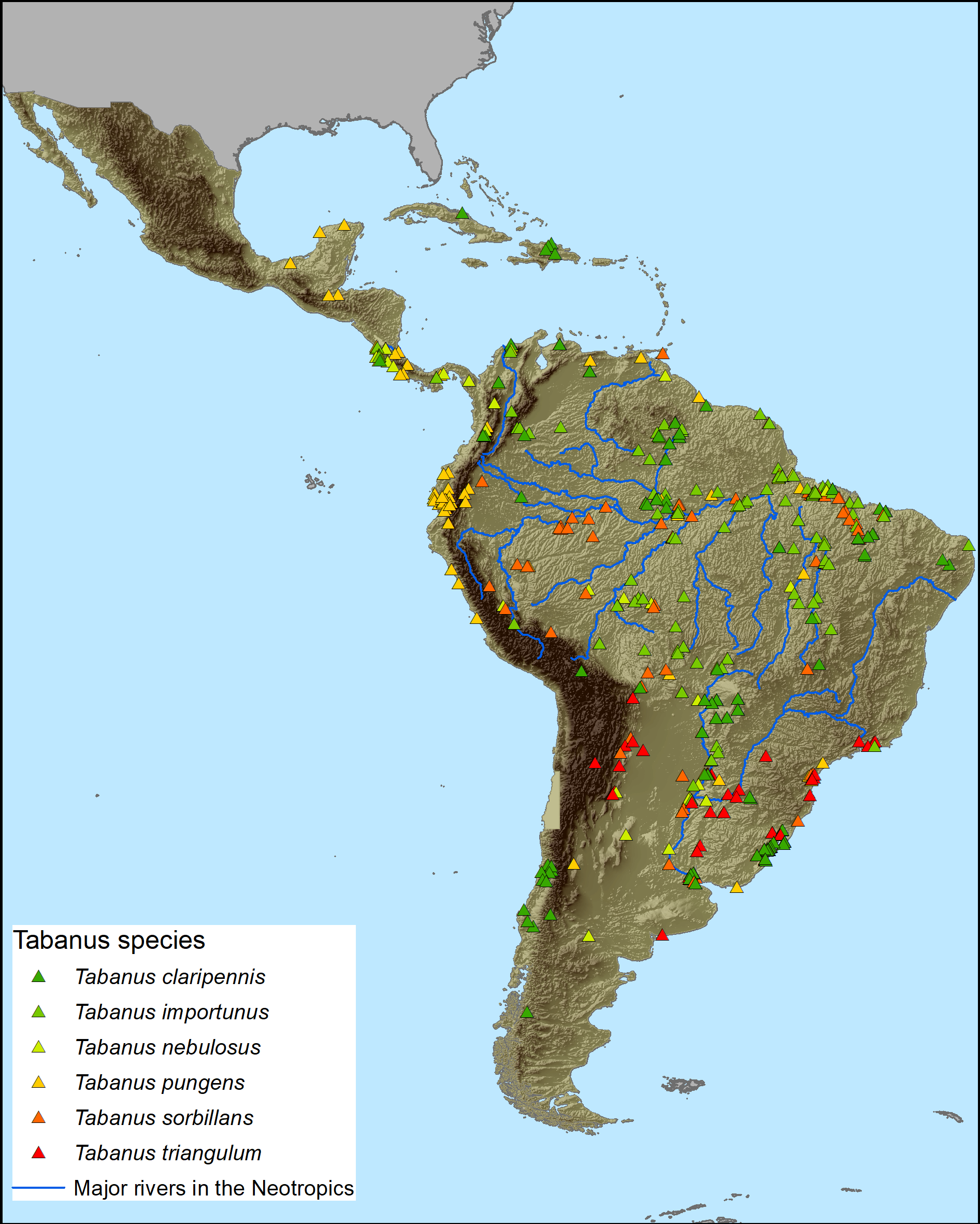


Supplementary file 3b. *Tabanus* species occurrences (black points) in the Neotropical region and the 100km buffer (M - pink circles) used in the models’ calibrations.

**
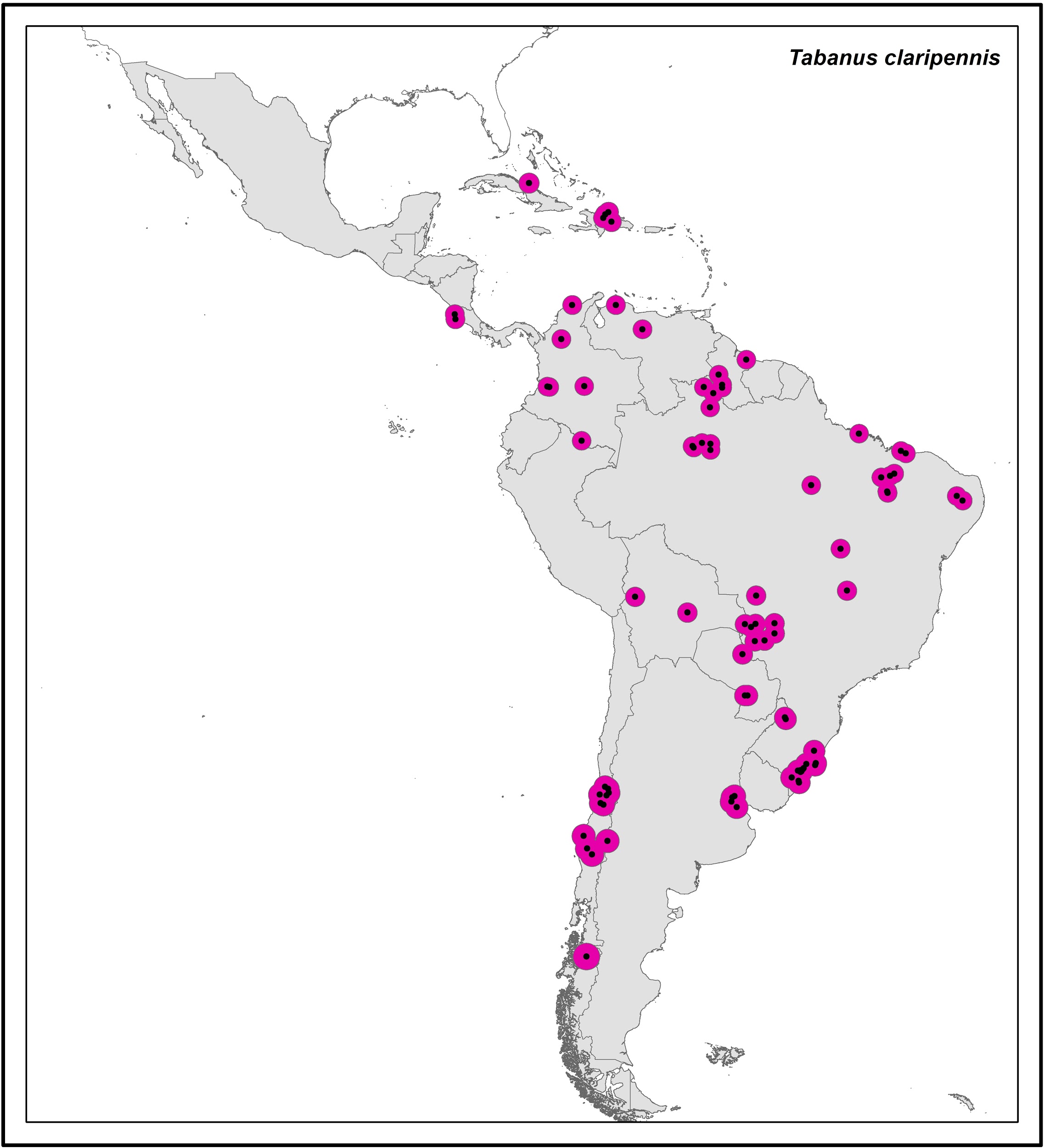
**

**
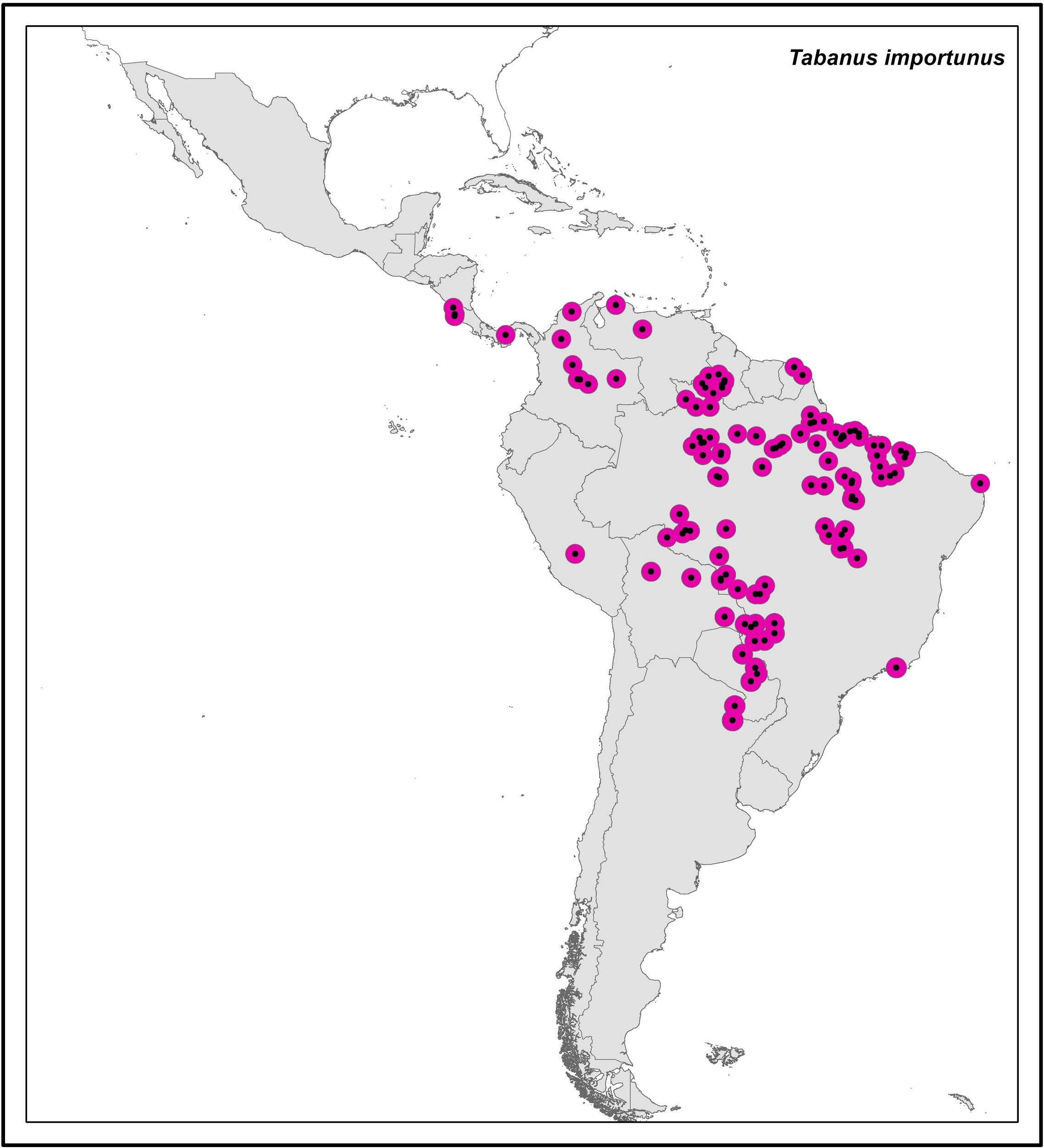
**


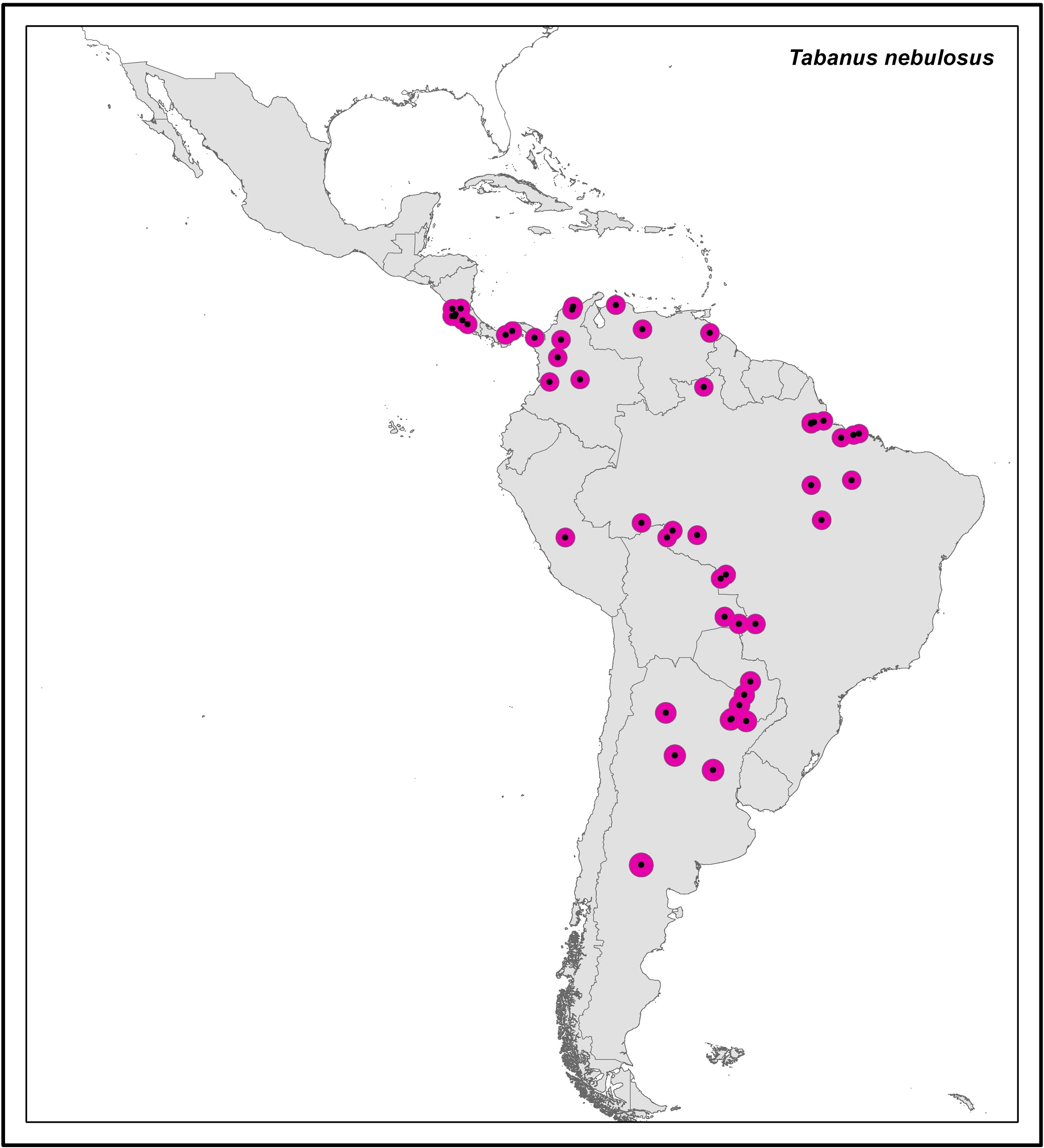


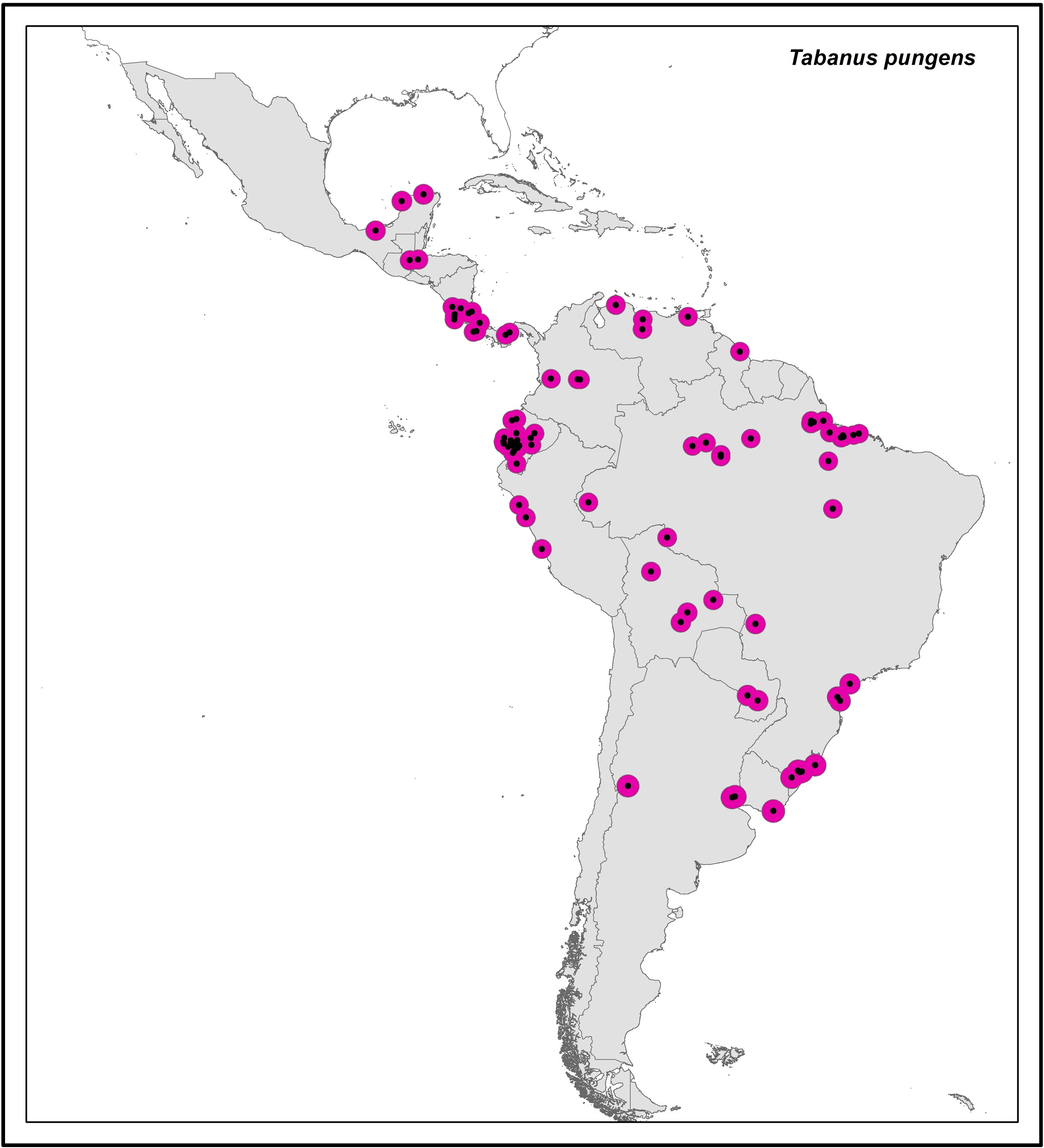


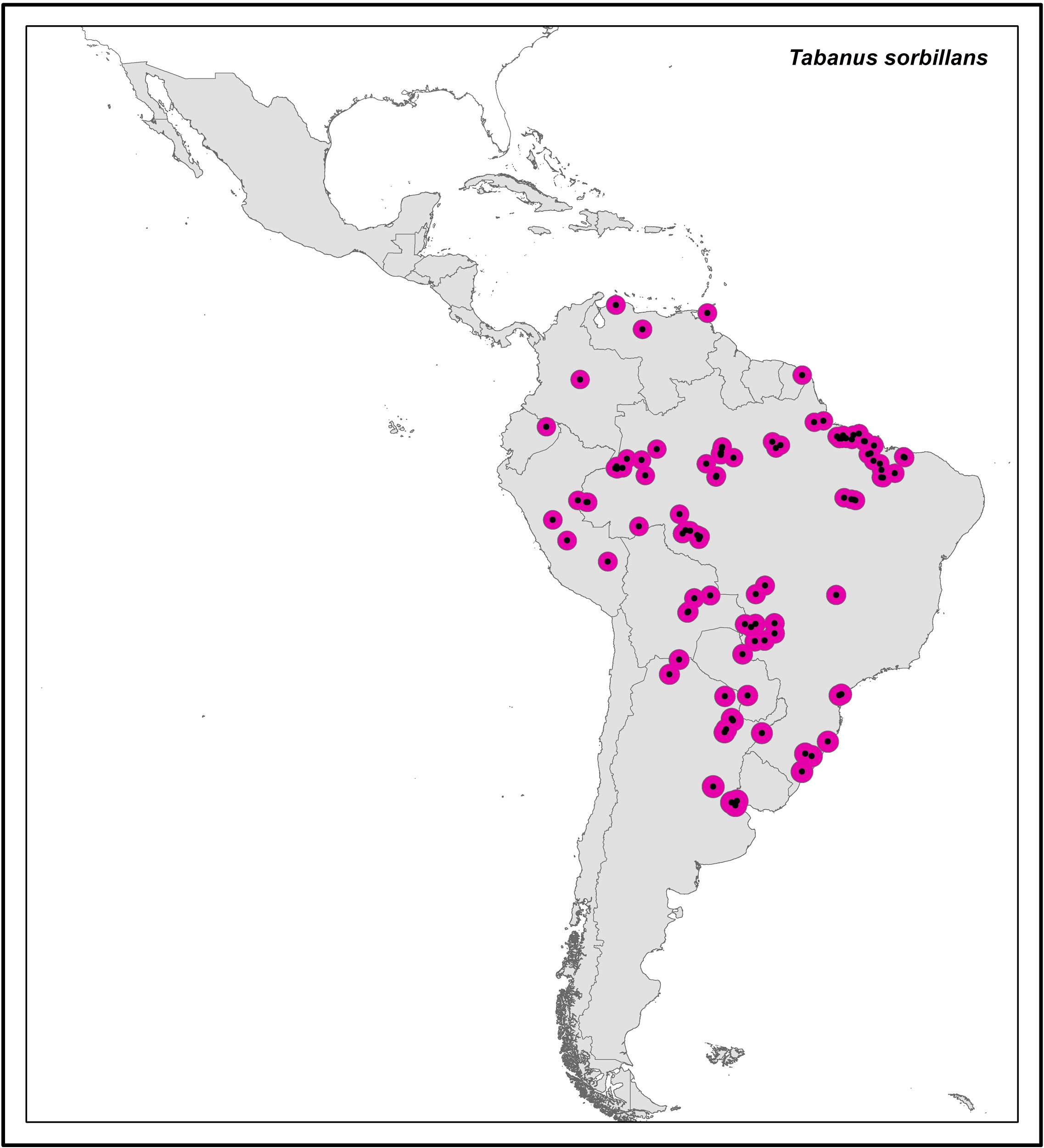


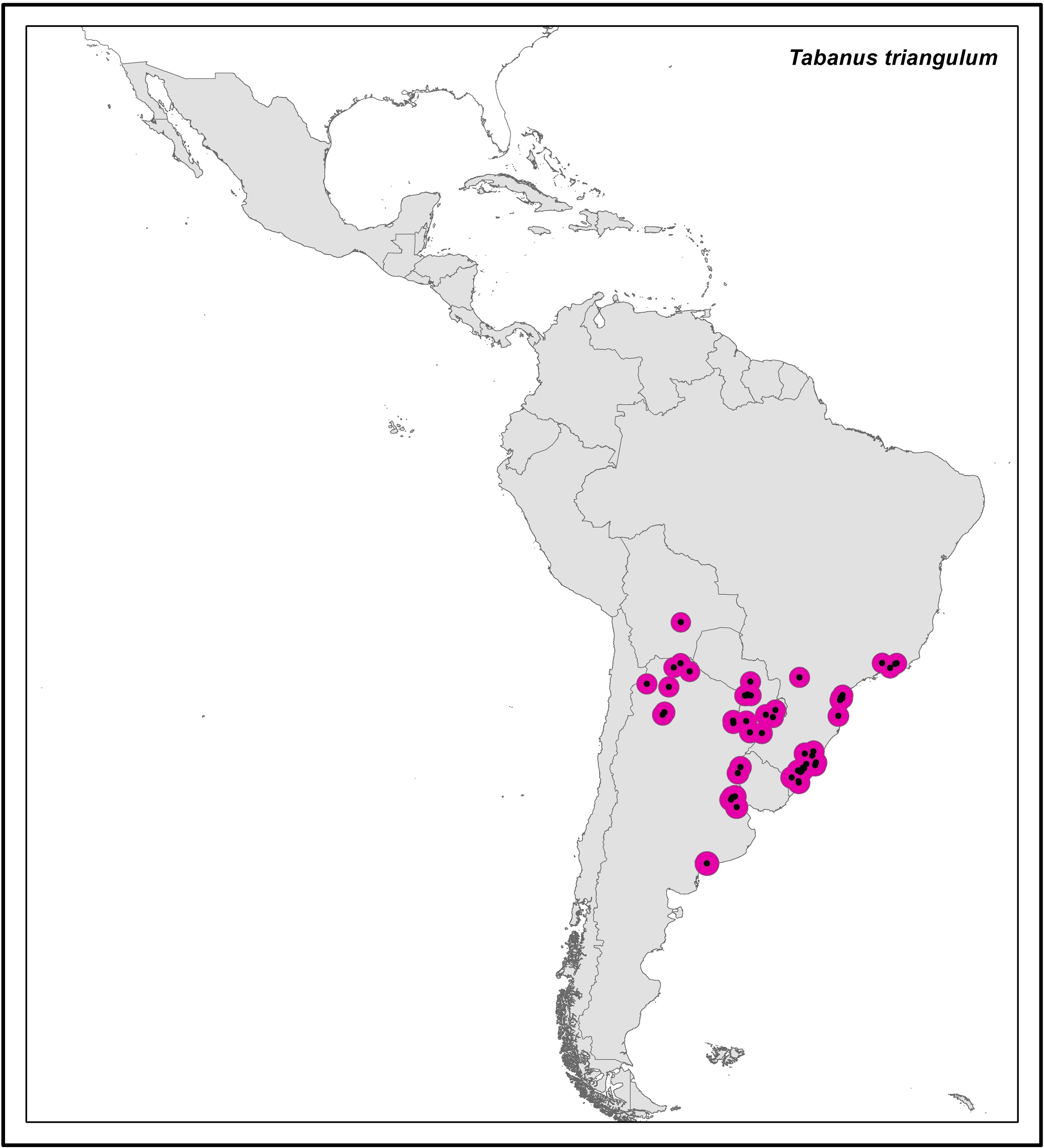

Supplement: Supplementary file 3 — Supplementary Material 3. Tabanus species occurrences (black points) in the Neotropical region and the 100-km buffer (M = pink circles) used in the calibration models. [file 13071_2025_6708_MOESM3_ESM.doc]
