# Supplementary material for: Spatial epidemiology of Tabanus (Diptera: Tabanidae) vectors of Trypanosoma
Source: Parasit Vectors. 2025 Apr 3;18:128. doi: 10.1186/s13071-025-06708-z (PMC11969902; doi:10.1186/s13071-025-06708-z)
Supplement: Supplementary file 4 — Supplementary Material 4. Models calibrated and evaluated for each Tabanus species, according to significance, performance, and low complexity. Final models selected according to these criteria are shown in the last column. [file 13071_2025_6708_MOESM4_ESM.doc]

Additional file 4. Models calibrated and evaluated for each *Tabanus* species, according to significance, performance, and low complexity. Final models selected according to these criteria are showed in the last column.

| ***Tabanus s*pecies** | **Candidate models** | **Statistically significant models (*P <* 0.05)** | **Performance (Omission rate < 0.05)** | **Final best models according to all criteria** |
| --- | --- | --- | --- | --- |
| *T. claripennis* | 54 | 39 | 6 | 1 |
| *T. importunus* | 54 | 8 | 4 | 1 |
| *T. nebulosus* | 54 | 32 | 3 | 3 |
| *T. pungens* | 54 | 41 | 3 | 1 |
| *T. sorbillans* | 54 | 21 | 1 | 1 |
| *T. triangulum* | 54 | 29 | 1 | 1 |
