# Supplementary material for: Spatial epidemiology of Tabanus (Diptera: Tabanidae) vectors of Trypanosoma
Source: Parasit Vectors. 2025 Apr 3;18:128. doi: 10.1186/s13071-025-06708-z (PMC11969902; doi:10.1186/s13071-025-06708-z)
Supplement: Supplementary file 5 — Supplementary Material 5. Best models selected by evaluation based on pROC (statistical significance), omission rate OR (performance), and AICc (complexity). All models were calibrated and projected using principal components from 15 climatic variables from the WorldClim Global Climate Database 1.4. [file 13071_2025_6708_MOESM5_ESM.doc]

Additional file 5. Best models selected by evaluated based on pROC (statistical significance) omission rate OR (performance), and AICc (complexity). All models were calibrated and projected using principal components from 15 climatic variables from WorldClim Global Climate Database 1.4.

| ***Tabanus s*pecies** | **Mean AUC Ratio** | **P value pROC** | **Omission rate at 5%** | **AICc** | **Delta AICc** | **Regul. multiplier** | **Feature**  **classes** |
| --- | --- | --- | --- | --- | --- | --- | --- |
| *T. claripennis* | 1.072 | 0 | 0.025 | 1751.884 | 0.000 | 3 | lq |
| *T. importunus* | 1.027 | 0 | 0.038 | 2554.681 | 0.000 | 10 | lq |
| *T. nebulosus* | 1.029 | 0 | 0.028 | 1084.049 | 0.000 | 0.5 | l |
|  | 1.026 | 0 | 0.028 | 1084.096 | 0.047 | 0.7 | l |
|  | 1.024 | 0 | 0.028 | 1084.195 | 0.146 | 1 | l |
| *T. pungens* | 1.099 | 0 | 0.049 | 1833.809 | 0.000 | 7 | lqp |
| *T. sorbillans* | 1.104 | 0 | 0.061 | 2258.754 | 0.000 | 5 | lqp |
| *T. triangulum* | 1.1 | 0 | 0.04 | 1023.95 | 0 | 3 | l |
