# Supplementary material for: Spatial epidemiology of Tabanus (Diptera: Tabanidae) vectors of Trypanosoma
Source: Parasit Vectors. 2025 Apr 3;18:128. doi: 10.1186/s13071-025-06708-z (PMC11969902; doi:10.1186/s13071-025-06708-z)

Additional file 6. MOP analysis of extrapolation risk from the calibration area under Neotropical region projection. Blue areas represent levels of similarity between calibration areas and the projection areas. Red values represent strict extrapolative areas.


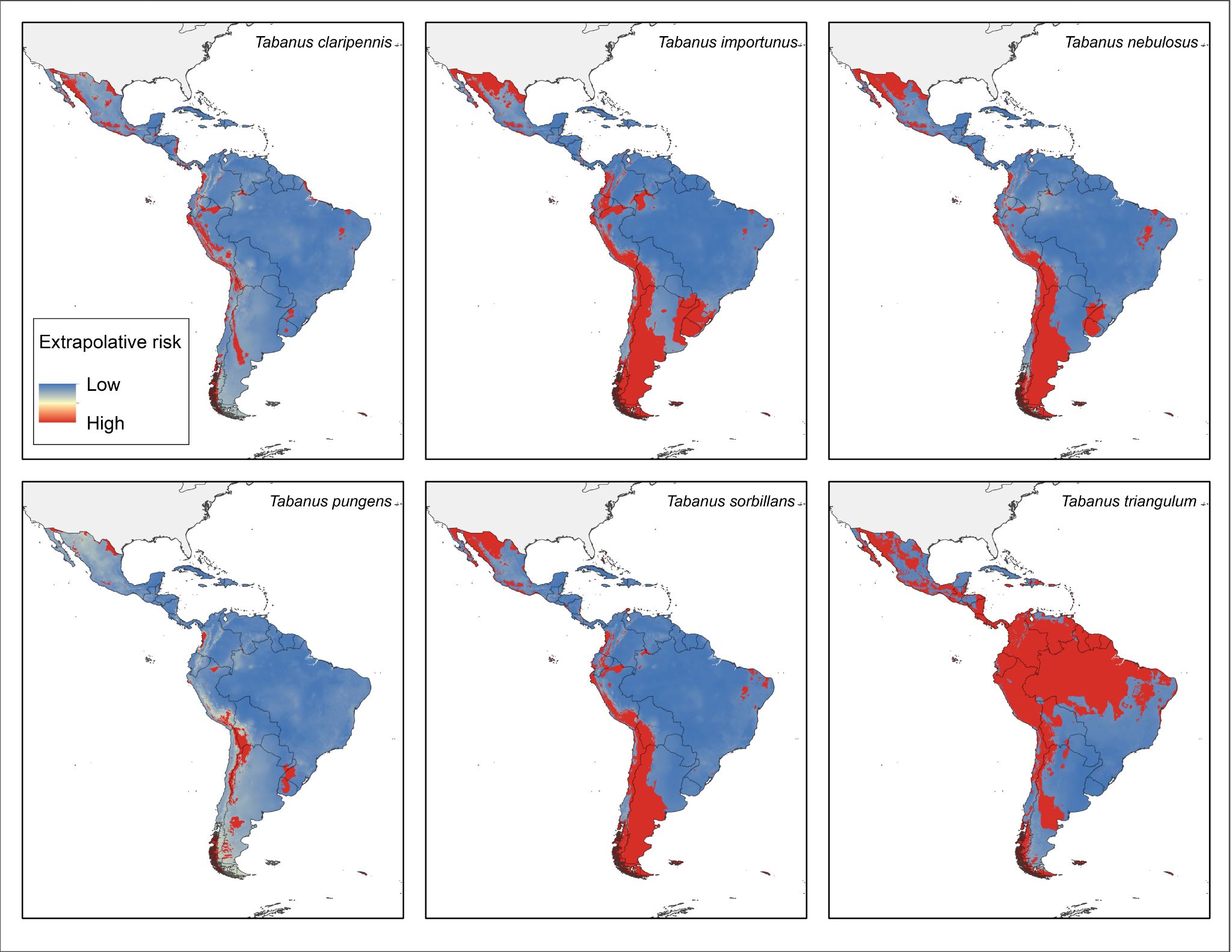

Supplement: Supplementary file 6 — Supplementary Material 6. MOP analysis of extrapolation risk from the calibration area under the Neotropical region projection. Blue areas represent levels of similarity between calibration areas and the projection areas. Red values represent strict extrapolative areas. [file 13071_2025_6708_MOESM6_ESM.doc]
